# Relationship among subjective responses, flavor, and chemical composition across more than 800 commercial cannabis varieties

**DOI:** 10.1186/s42238-020-00028-y

**Published:** 2020-07-17

**Authors:** Alethia de la Fuente, Federico Zamberlan, Andrés Sánchez Ferrán, Facundo Carrillo, Enzo Tagliazucchi, Carla Pallavicini

**Affiliations:** 1 Buenos Aires Physics Institute (IFIBA) and Physics Department, University of Buenos Aires, Buenos Aires, Argentina; 2 Institute of Cognitive and Translational Neuroscience (INCYT), INECO Foundation, Favaloro University, Buenos Aires, Argentina; 3 National Scientific and Technical Research Council (CONICET), Buenos Aires, Argentina; 4 National University of Tucumán, San Miguel de Tucumán, Argentina; 5 Applied Artificial Intelligence Lab, ICC, CONICET, Buenos Aires, Argentina; 6 Grupo de Investigación en Neurociencias Aplicadas a las Alteraciones de la Conducta, FLENI-CONICET, Buenos Aires, Argentina

**Keywords:** Cannabis, Cultivars, Terpenes, Cannabinoids, Flavour, Chemotypes, Subjective reports

## Abstract

**Background:**

Widespread commercialization of cannabis has led to the introduction of brand names based on users’ subjective experience of psychological effects and flavors, but this process has occurred in the absence of agreed standards. The objective of this work was to leverage information extracted from large databases to evaluate the consistency and validity of these subjective reports, and to determine their correlation with the reported cultivars and with estimates of their chemical composition (delta-9-THC, CBD, terpenes).

**Methods:**

We analyzed a large publicly available dataset extracted from Leafly.com where users freely reported their experiences with cannabis cultivars, including different subjective effects and flavour associations. This analysis was complemented with information on the chemical composition of a subset of the cultivars extracted from Psilabs.org. The structure of this dataset was investigated using network analysis applied to the pairwise similarities between reported subjective effects and/or chemical compositions. Random forest classifiers were used to evaluate whether reports of flavours and subjective effects could identify the labelled species cultivar. We applied Natural Language Processing (NLP) tools to free narratives written by the users to validate the subjective effect and flavour tags. Finally, we explored the relationship between terpenoid content, cannabinoid composition and subjective reports in a subset of the cultivars.

**Results:**

Machine learning classifiers distinguished between species tags given by “*Cannabis sativa”* and “*Cannabis indica”* based on the reported flavours*: <*AUC> = 0.828 ± 0.002 (*p* < 0.001); and effects: <AUC> = 0.9965 ± 0.0002 (*p* < 0.001). A significant relationship between terpene and cannabinoid content was suggested by positive correlations between subjective effect and flavour tags (*p* < 0.05, False-Discovery-rate (FDR)-corrected); these correlations clustered the reported effects into three groups that represented unpleasant, stimulant and soothing effects. The use of predefined tags was validated by applying latent semantic analysis tools to unstructured written reviews, also providing breed-specific topics consistent with their purported subjective effects. Terpene profiles matched the perceptual characterizations made by the users, particularly for the terpene-flavours graph (Q = 0.324).

**Conclusions:**

Our work represents the first data-driven synthesis of self-reported and chemical information in a large number of cannabis cultivars. Since terpene content is robustly inherited and less influenced by environmental factors, flavour perception could represent a reliable marker to indirectly characterize the psychoactive effects of cannabis. Our novel methodology helps meet demands for reliable cultivar characterization in the context of an ever-growing market for medicinal and recreational cannabis.

## Background

*Cannabis plants* have been used for millennia around the world, (Mechoulam 2019; Russo 2011; Russo et al. 2008). Historically, they had been classified into two separate species (Indica and Sativa s.p.) presenting different botanical characteristics, such as height, leaf width, and others (Bonini et al. 2018; Lamarck 1785). While some contemporary authors have supported this categorization via chemical analysis (Hillig 2004; Hillig and Mahlberg 2004), the most accepted position is the elimination of this classification (Piomelli and Russo 2016).

In recent times, the commercialization of cannabis has increased dramatically, leading to the appearance of several commercial cultivars in the absence of agreed standards. It is important to note that these “recreational users” (as opposed to medical users) sometimes also seek therapeutic effects when consuming the plant. This applies not only to cannabis users but also to those of lavender, yerba mate, coffee, ginger roots or any other plants which are used with certain expected therapeutic effects (e.g. soothing, stimulant, etc.) beyond their aromas and flavours.

Market growth for cannabis has been dramatic in some countries; for instance, in the United States sales reached $6.7 billion in 2016, with 30% growth year-over-year, representing the second largest cash crop, with total worth over $40 billion (Adams 2019; Robinson 2017). These sudden changes created novel problems for users, as cannabis cultivators transition towards legal business models, yet without a world-wide standard for their products. Moreover, “species” (e.g. “indica”, “sativa”) and “cultivar names” might not be representative of the underlying chemovar, yet these categories are still frequently used in commercial contexts in spite of their doubtful botanic validity (Jikomes and Zoorob 2018; Piomelli and Russo 2016), this presents a problem for users who cannot rely on the provided name to guarantee the desired effect. Cannabis dispensaries offer dry cannabis flowers or buds (Gilbert and DiVerdi 2018), extracts and essential oils (Permanente and Care 2008) and various edibles (Weedmaps n.d.); however, since in most countries these products remain illegal, there are no international agreements to regulate their quality or chemical content. To fulfill this contemporary demand, websites and commercial repositories (such as Leafly.com) have begun to gather a large number of user reports. However, comprehensive analyses of the validity of these reports and their correlation with chemical composition of the consumed plants are still lacking.

Understanding the relationship between user reports and the chemical composition of cannabis is very relevant, since the development of standards could be further complicated by the heterogeneous composition of the plants. Cannabis contains over 400 compounds, including more than 60 cannabinoids, the main active molecules being tetrahydrocannabinol (delta-9-THC) and cannabidiol (CBD) (Pollastro et al. 2018). These two cannabinoids were often considered the only chemicals involved in the medicinal properties and psychoactive effects associated with cannabis, and remain the only ones screened when evaluating cultivar chemotypes (De Meijer et al. 2009; Fetterman et al. 1971; Hazekamp et al. 2016; Nie et al. 2019; UNODC 1968). However, increasing evidence supports the relevance of terpenes and terpenoids, molecules responsible for the flavour and scent of the plants, both as synergetic to cannabinoids and as active compounds by themselves (Henry 2017; Hillig 2004; Nuutinen 2018; Russo 2011). Flavours have predictive value at cultivar level (Gilbert and DiVerdi 2018) that may be complementary to the quantification of THC and CBD content (Jikomes and Zoorob 2018). Terpenes are widely used as biochemical markers in chemosystematics studies to characterize plant samples due to the fact that they are under strong genetic control and relatively unaffected by environmental factors (Aizpurua-Olaizola et al. 2016; Casano et al. 2011; Hillig 2004). Cannabinoid content, on the other hand, can vary greatly among generations of the same strain, and also due to the sex, age and part of the plant (Fetterman et al. 1971; Hazekamp et al. 2016).

In this work, we combined different sources of data for the characterization of commercial cannabis cultivars, linking both self-reports of psychoactive effects and flavour profiles with information obtained from experimental assays of cannabinoid and terpene content. Our analysis comprised 887 different cultivars and was based on a large sample (> 100.000) of user reviews publicly available at the website Leafly (www.leafly.com). The reports contained unstructured written reviews of experiences for each commercial strain, as well as structured tags indicating flavour profiles and subjective effects. As for cultivar categorization, we used the “indica“, “sativa “and “hybrid “labels, since they were the ones provided in the Leafly dataset.

We explored the following four hypotheses: 1) supervised and unsupervised machine learning algorithms can group cultivars into clusters of similar breeds based on subjective effect tags, but also based on flavour profile tags, 2) certain pairs of effect and flavour tags are correlated across cultivars, implying potential association between terpene and cannabinoid content 3) unstructured written reports contain information consistent with the tags, and the detection of recurrent topics in the reports matches the known effects and uses of different cannabis breeds, and 4) terpene and cannabinoid profiles are consistent with the perceptual characterizations made by the users. In particular, since terpene content is a major factor influencing cannabis scent and flavor, we expect a significant correlation of terpene profile with tags. We stress that our work does not include data specific pathological treatments (e.g. epilepsy, melanoma, multiple sclerosis, etc. [Bonini et al. 2018]), and focused only on a large sample of recreational users of commercial cannabis.

## Methods

### User reported data

Data corresponding to > 1.200 cannabis cultivars was accessed and downloaded from Leafly (www.leafly.com) (August 2018). Leafly is presently the largest cannabis website in the world wide web, allowing users to rate and review different cultivars of cannabis and their dispensaries. Sets of predefined tags could be used to rate subjective effects (e.g. “aroused”, “creative”, “euphoric”, “relaxed”, “paranoid”). Here, subjective effects stand for those effects that impact primarily on subjective experience, and are determined by the direct reports made by the users. Flavours (e.g. “apple”, “coffee”, “flowery”, “apricot”, “vanilla”) are assigned to cultivars via crowdsourcing, together with a large number (> 100.000) of unstructured written reviews.

Cultivars with less than 10 reviews were discarded (Martial et al. 2019; Sanz and Tagliazucchi 2018; Zamberlan et al. 2018), resulting in 887 cultivars included in this study. We also verified that single user reports per “strain” represented less than 20% of the total reports in 99% of the data and performed extensive user/report statistical descriptions (Supplementary Table 1) Each cultivar was arbitrary classified by Leafly users as “indica“, “sativa “or “hybrid“. Users associate cultivars with tags indicating flavours (48 different tags) and effects (19 different tags).

It should be noted that biases can appear in crowdsourced data when a large proportion of the reports comes from a small group of users, hence violating the independence assumption. We studied this possibility by computing and visualizing the cumulative histogram of reports per subject per cultivar (see supplementary Fig. 1). Only 10% of the studied cultivars presented more than 10% of their reports given by a single user, which represents one order of magnitude of difference between the total dataset size and the potentially non-independent data. Moreover, this was reduced to only 0.6% of varieties with more than 20% reports by a single user, which suggests that subject identity impacts a very reduced sample of the dataset. Details on all included cultivars, flavour and effect tags are presented in Table 1 and in additional information [see Additional file 1].

**Table 1:** Counts of user reports by cultivar

		Reports				Users					Reports by cultivar by user		Reports by cultivar	
Partition	N cultivars	N tot	% tot	N.I.	%N.I.	N users	% =1	% > 1	% > 2	% > 3	%median (IQR,Skew)	%max	%median (IQR,Skew)	max
All reports	887	100.901	100	983	1	43.925	57	43	21	12	0.61 (1.2,3.44)	40	54 (98.5,3.64)	1456
Sativa	171	18.193	18	211	1	10.824	67	33	12	6	0.74 (1.36,2.74)	18	52 (72,4.15)	1373
Indica	265	30.977	31	316	1	16.803	54	37	15	8	0.59 (1.13,3.95)	38	61 (106,4.22)	1456
Hybrid	451	51.731	51	456	1	26.259	61	39	17	10	0.58 (1.18,3.4)	40	52 (100.5,2.87)	1225

Summaries of reports, users, reports by cultivar, and reports by cultivar by user (August 2018). N.I.: Anonymous; tot: total; IQR: Interquartile range; Skew calculated using R defaults, as in DeCarlo (1997)

### Effect and flavour tags

Given a cultivar *s* with *n* reviews, we considered for the i-th review the vectors ${E}_i=\left({e}_1^i,\dots, {e}_{19}^i\right)$ and ${F}_i=\left({f}_1^i,\dots, {f}_{48}^i\right)$, where ${e}_j^i=1$ if the tag for the j-th effect appeared in the i-th review, and ${e}_j^i=0$ otherwise. The ${f}_j^i$ were defined analogously, but based on the flavour tags. Next, the cultivar was identified with the vectors $\mathrm{E}\left(\mathrm{s}\right)=\frac{1}{\mathrm{n}}{\sum}_{\mathrm{i}=1}^{\mathrm{n}}{\mathrm{E}}_{\mathrm{i}}=\frac{1}{\mathrm{n}}{\sum}_{\mathrm{i}=1}^{\mathrm{n}}\left({\mathrm{e}}_1^{\mathrm{i}},\dots, {\mathrm{e}}_{19}^{\mathrm{i}}\right)$ and $\mathrm{F}\left(\mathrm{s}\right)=\frac{1}{\mathrm{n}}{\sum}_{\mathrm{i}=1}^{\mathrm{n}}{\mathrm{F}}_{\mathrm{i}}=\frac{1}{\mathrm{n}}{\sum}_{\mathrm{i}=1}^{\mathrm{n}}\left({\mathrm{f}}_1^{\mathrm{i}},\dots, {\mathrm{f}}_{48}^{\mathrm{i}}\right)$, representing the probability that each subjective effect and flavour tag was used in the description of the corresponding “strain“.

### Network and modularity analysis

Given two cultivars *s*_1_ and *s*_2_, they were represented in the effect / flavour network by nodes linked with a connection weighted by the value of the non-parametric Spearman correlation between vectors *E*(*s*_1_) and *E*(*s*_2_) / *F*(*s*_1_) and *F*(*s*_2_), respectively. To find sub-networks with dense internal connections and sparse external connections (i.e. modules), the Louvain agglomerative algorithm (Blondel et al. 2008) was applied to maximize Newman’s modularity using a resolution parameter γ = 1 .

To visualize the resulting networks, we used the ForceAtlas 2 layout included in Gephi (Bastian et al. 2009) (https://gephi.org/). ForceAtlas 2 represents the network in two dimensions, modeling the link weights (i.e. Spearman correlations) as springs, and the nodes as point charges of the same sign. The attraction is then computed using Hooke’s law (Hooke 1678) and the repulsion using Coulomb’s law (Coulomb 1785). A detailed explanation can be found in the additional file 1, section 2.

### Effect-flavour correlation analysis

For all cultivars, the effect and flavour frequency vectors can be summarized as matrices *E*_*is*_ and *F*_*is*_ of size 887 × 19 and 887 × 48, respectively, indicating the probability of observing the i-th effect / flavour tag in cultivar *s*. To investigate associations between subjective effect and flavour tags, we computed all 19 × 48 = 912 non-parametric Spearman correlations between all possible pairs of columns from $\hat{E}$ and $\hat{F}$. We follow the notation where $\hat{A}$ refers to a matrix and *A*_*ij*_ to a particular matrix entry. Since some effect and flavour tags appeared very sparsely, we only considered pairs of cultivars for which at least one report included the given flavour / effect tag (i.e. we excluded columns of zeros from the correlation analysis). As an example, if a given “strain” with 10 reports neither included the ‘Blue cheese’ flavour, nor the ‘Uplifted’ effect, this category pair did not include this variety in the correlation analysis. This restricted the analysis only to the valid or more representative cultivars.

### Random forest classifiers

To investigate whether effect and flavour tags could discriminate between different cannabis “species“ tags assigned by users, we trained and evaluated (5-fold stratified cross-validation) machine learning classifiers to distinguish the 265 “indicas” from the 171 “sativas” in the dataset, using as features the corresponding E(s) and F(s) vectors for each cultivar *s*.

Classifiers were based on the random forest algorithm (Hastie 2009; James et al. 2013), as implemented in scikit-learn (https://scikit-learn.org/), and a detailed explanation can be found in the additional file 1, section 2. This algorithm builds upon the concept of a decision tree classifier, in which the samples are iteratively split into two branches, depending on the values of their features. For each feature, a threshold is determined so that the samples are separated to maximize a metric of the homogeneity of the class labels assigned to the resulting branches. The algorithm stops when a split results in a branch where all the samples belong to the same class, or when all features are already used for a split. This procedure is prone to overfitting, because a noisy or unreliable feature selected early in the division process could bias the remaining part of the decision tree. To attenuate this potential issue, the random forest algorithm creates an ensemble of decision trees based on a randomly chosen subset of the features. After training each tree in the ensemble, the probability of a new sample belonging to each class was determined by the aggregated vote of all decision trees. We divided our dataset into 5 equal parts and used 4 parts to train the model and the remaining part for testing (5-fold stratified cross-validation). This procedure results in a robust tool for sensitivity analysis (Ermagun et al. 2020; Kamalov 2019), which is valuable in the case of our crowdsourced data. We trained random forests using 1.000 decision trees and a random subset of features of size equal to the rounded square root of the total number of features. The quality of each split in the decision trees was measured using Gini impurity, and the individual trees were expanded until all leaves were pure (i.e. no maximum depth was introduced). No minimum impurity decrease was enforced at each split, and no minimum number of samples were required at the leaf nodes of the decision trees. All model hyperparameters are detailed in the scikit-learn documentation (https://scikit-learn.org/).

To assess the statistical significance of the output, we trained and evaluated 1.000 independent random forest classifiers using the same features but after scrambling the class labels. We then constructed an empirical *p*-value by counting the number of times that the accuracy of the classifier based on the scrambled labels exceeded that of the original classifier. The accuracy of each individual classifier was determined by the area under the receiver operating characteristic curve (AUC).

### Natural language processing of written unstructured reports

Text preprocessing was performed using the Natural Language Toolkit (NLTK, http://www.nltk.org/) in Python 3.4.6. The following steps were applied: 1) discarding all punctuation marks (word repetitions allowed) and splitting into individual words, 2) word conversion to the root from which the word is inflicted using NLTK (i.e. lemmatization), 3) conversion to lowercase, 4) after lemmatization, words containing less than two characters were discarded (Sanz and Tagliazucchi 2018).

To quantitatively explore the semantic content of the reports we used Latent Semantic Analysis (LSA) (Landauer et al. 1998; Martial et al. 2019; Sanz and Tagliazucchi 2018) [see additional file 1, section 2] over all combined cultivar reports (*N* = 100.901) in the subsamples of “indicas“(30.977 reports from 265 cultivars) and “sativas“(18.193 reports from 171 cultivars). For this, we constructed a matrix *A*_*wj*_ containing in its w,j position the weighted frequency of the w-th term in the combined reports of the j-th strain. The weighted frequency (tf-idf weighting) was computed as ${f}_{wd}=\mathit{\log}\frac{\left|D\right|}{f_{dw}}$, where *f*_*wd*_ represents the frequency of word *w* in document *d*, |*D*| indicates the total number of documents, and *f*_*dw*_ is the fraction of documents in which word *w* appears. To avoid very common / uncommon words, we kept only those appearing in more than 5% / less than 95% of the documents, respectively.

LSA was applied to reduce the rank of *A*_*wj*_, thus reducing its sparsity and identifying different words by semantic context. For this purpose, the matrix was first decomposed using Singular Value Decomposition (SVD) into the product of three matrices (Huang and Narendra 2008) as $\hat{A}=\hat{U}\times \hat{S}\times \hat{W}$, where $\hat{U}$ contains the matrix eigenvectors, $\hat{S}$ is a diagonal matrix containing the ordered eigenvalues of $\hat{A}{\hat{A}}^T$, and $\hat{W}$ contains the eigenvectors of ${\hat{A}}^T\hat{A}$. To reduce the dimensionality of the semantic space, only the first 50 singular values of $\hat{S}$ were retained, yielding the truncated diagonal matrix ${\hat{S}}_{50}$. From this matrix, the rank reduced matrix was computed as ${\hat{A}}_{50}={\hat{U}}_{50}\times {\hat{S}}_{50}\times {\hat{W}}_{50}$. ${\hat{A}}_{50}$ is here referred to as the reduced rank word-document matrix. By computing the Spearman correlation coefficient between the columns of ${\hat{A}}_{50}$ it is possible to estimate the semantic similarity between the written reports associated with pairs of cultivars. Alternatively, this can be conceptualized as a network, where nodes correspond to cultivars and links are weighted by the semantic similarity between their associated sets of reports. The choice of rank 50 was validated by investigating the stability of the number of communities and the modularity values detected in this network using the Louvain algorithm. This validation is included as an additional figure (see Additional file 1).

### Principal component analysis and topic detection

To reduce the term-document matrix into a smaller number of components capturing topics appearing recurrently in the corpus of reports, we performed a principal component analysis (PCA) using MATLAB SVD decomposition algorithm (see additional file 1, section 2). We analyzed the first five components, i.e. the components explaining most of the variance. Each component consisted of a combination of words present in the vocabulary, and the coefficients were used to represent the importance of the words.

### Association between tags and unstructured written reports

To provide an example of the relationship between the reported effect tags and the unstructured written reports, we performed the LSA analysis on two cultivars with a large number of reports: a cultivar representative of the “sativa” tag (Super lemon haze, 1.373 reports), and another representative of the “indica” (Blueberry, 1.456 reports). In this case, the matrix *A*_*ij*_ was constructed so that rows represented unique terms in the vocabulary, and columns represented individual reports (i.e. the reports were not pooled for each “strain”). We then performed PCA for each of the cultivars and retained the first 25 terms included in the first five components, comparing them afterwards to the most frequently reported effect tags for each “strain”. The semantic comparison was performed using the Datamuse API (www.datamuse.com), a word-finding engine based on word2vec (Minarro-Gimenez et al. 2014), an embedding method using shallow neural networks to map words into a vector space with the constraint that words appearing in similar contexts are also close in the vector space embedding (see additional file 1, section 2). We applied this tool to measure the mean distance of each tag to the words in each component, and then compared this distance to the one obtained using random English words extracted from www.wordcounter.net/random-word-generator.

### Terpene and cannabinoid data

Cannabinoid and terpene profiles of commercial samples of cannabis cultivars were manually downloaded from the PSI Labs webpage (psilabs.org, retrieved in August 2018). PSI Labs is an ISO 17025 accredited Safety Compliance Facility, based in Ann Arbor, Michigan state, licenced by Michigan State for testing medical and recreational cannabis samples. As detailed in the website, cannabinoid content was assessed using high-performance liquid chromatography (HPLC) with a diode-array detector (DAD), and terpene content was assessed using gas chromatography–mass spectrometry (GC-MS). This website contains a large number (> 1.600) of test results, with mass spectrometry profiles for 14 cannabinoids and 33 terpenes. We downloaded test results corresponding to cultivars with more than 10 reports in Leafly, yielding a sample of 443 test results from 183 different cultivars. We discarded terpenes and cannabinoids that were reported in less than three cultivars, resulting in profiles comprising 10 cannabinoids and 25 terpenes.

## Results

**Fig. 1:**
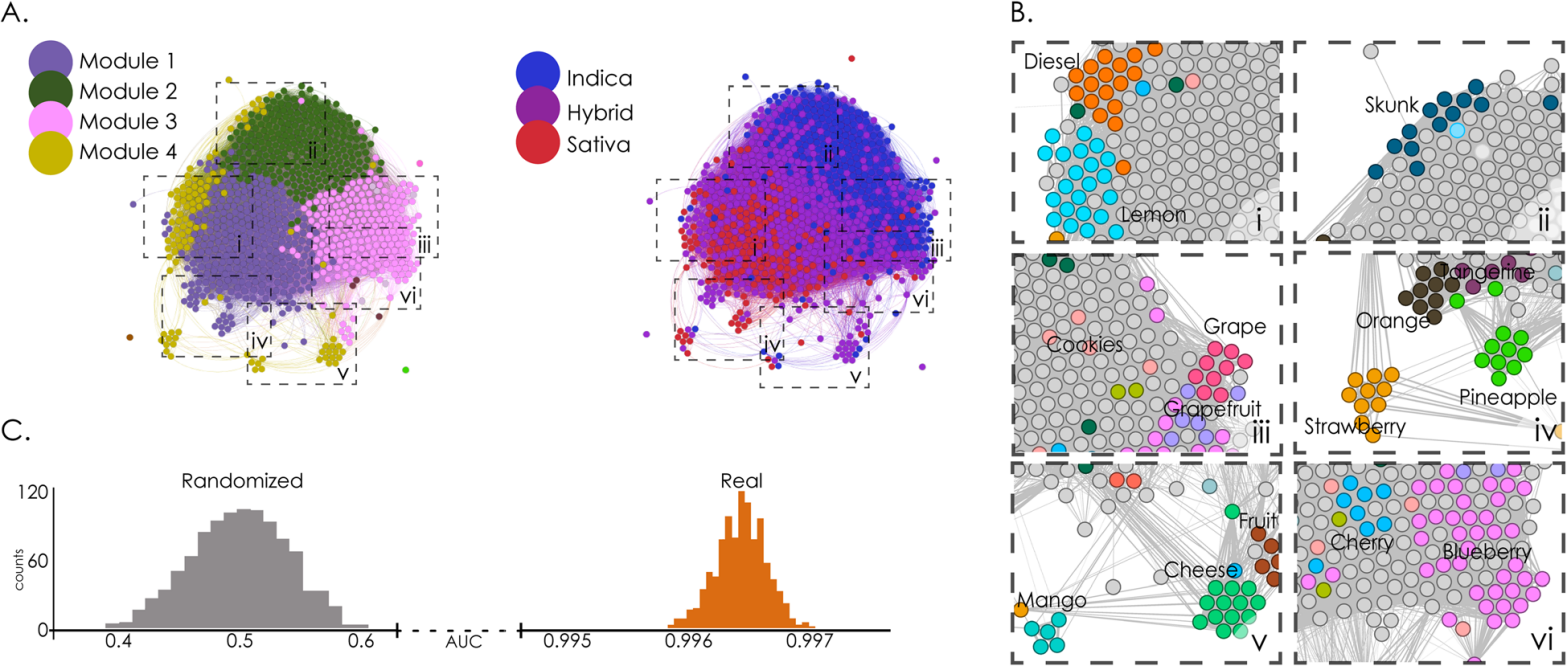
Analysis of the effect similarity network allowed supervised and unsupervised cannabis species classification. A. Effect similarity networks, with nodes representing cultivars and spatial proximity reflecting the Spearman correlation of the corresponding effect frequency vectors. The left panel is color-coded based on the results of modularity optimization using the Louvain algorithm, while the right panel is color-coded based on species tags (“indica”, “sativa”, “hybrids”). B. Subpanels zooming into different regions of the networks to show that cultivars sharing naming conventions were grouped together. C. Histogram of AUC values obtained over 1000 iterations of random forest classifiers using the effect frequency vectors as features and species tags (“indica” and “sativa”) as labels. “Randomized” indicates counts of AUC values obtained after randomly shuffling the sample labels

Following our first hypothesis, we applied supervised and unsupervised machine learning algorithms to the subjective effect tags and recovered cultivar clustering into similar breeds. Also, metrics of cultivar similarity based on self-reported effects allowed machine learning classification into the species tag as *Cannabis “sativa”* and *Cannabis “indica”.* This network is represented in Fig. 1a using the ForceAtlas 2 layout, which increases the proximity of nodes with strong connections. The Louvain algorithm produced a partition with modularity Q = 0.264 and a total of 18 modules, of which the largest five contained ≈ 98% of all cultivars, see Supplementary information 5. The network color-coded by species tags showed a clear separation of “indicas” and “sativas”, with cultivars labeled as “hybrids” located in between. Module 1 contained most of the “sativa” cultivars, while “indicas” and “hybrids” appeared distributed across the other modules.

Strains with names indicative of particular flavours clustered together in this network. Sub-panels I-VI (Fig. 1b) zoom into different regions of the network, showing that cultivars with similar naming conventions were strongly connected in the effect similarity graph. This was the case for lemons and diesels (I), skunks (II), grapes, cherries and berries (III), pineapples, oranges and strawberries (IV), fruits, cheeses and mangos (V), and blueberries (VI). We also described these groups by their general category, e.g. “lemons”, “grapefruits”, “strawberries” were labeled “fruits”. This grouping suggests the presence of correlations between effect and flavour tags, a possibility which is explored in the following sections.

Using the effect tag frequency vectors *E*(*s*) as features in a random forest classifier trained to distinguish “indicas” from “sativas” tags resulted in a highly accurate classification (Fig. 1c), with <AUC> = 0.9965 ± 0.0002 (mean ± standard deviation [STD], *p* < 0.001).

**Fig. 2:**
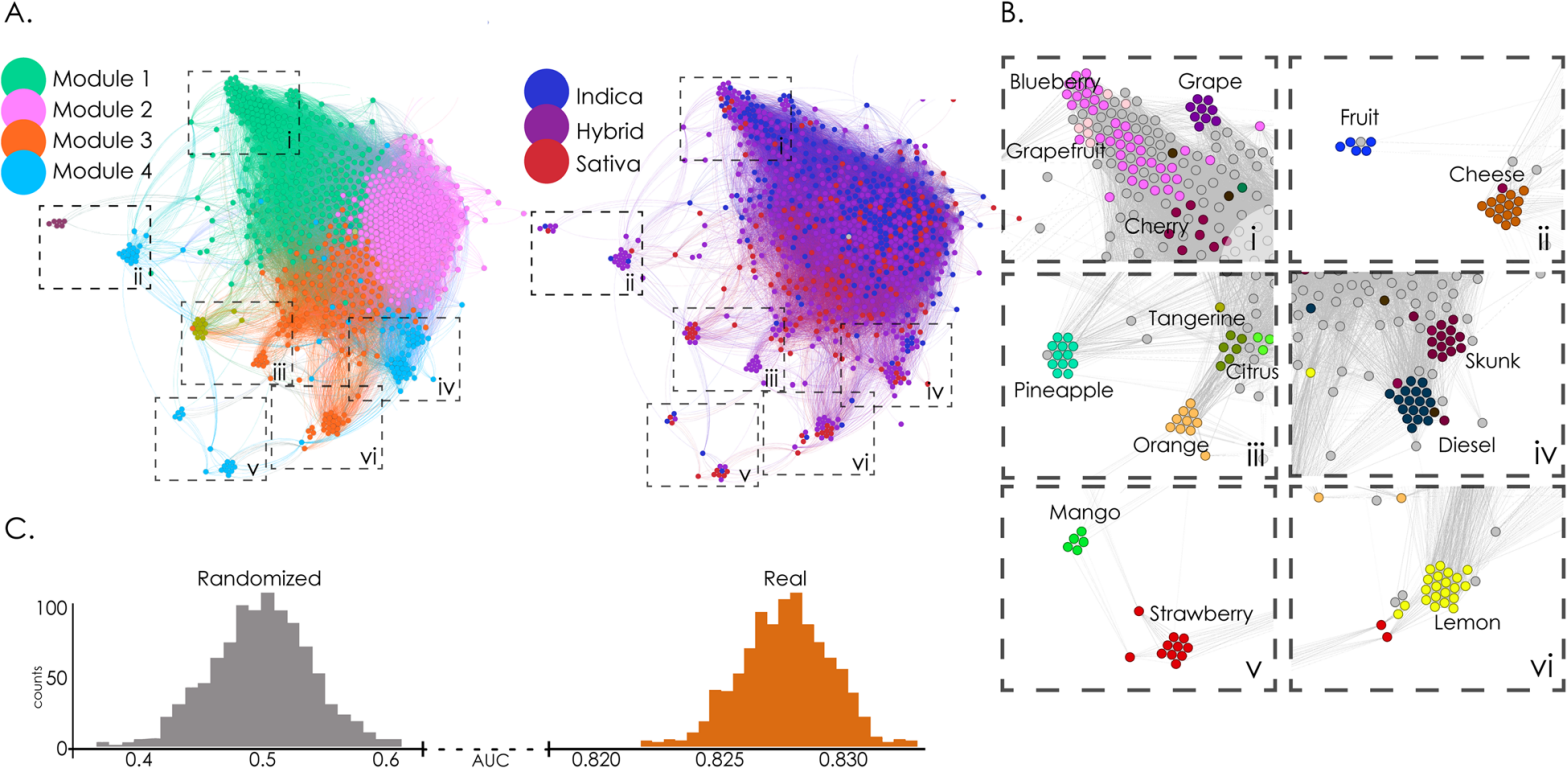
Analysis of the flavour similarity network allowed supervised and unsupervised cannabis species tag classification. A. Flavour similarity networks, with nodes representing cultivars and spatial proximity reflecting the Spearman correlation of the corresponding effect frequency vectors. The left panel is color-coded based on the results of modularity optimization using the Louvain algorithm, while the right panel is color-coded based on species tags (“indica”, “sativa”, “hybrid”). B. Subpanels zooming into different regions of the networks show that cultivars sharing naming conventions and flavours were grouped together. C. Histogram of AUC values obtained from 1000 iterations of random forest classifiers using the flavour frequency vectors as features and species (“indica” and “sativa”) as labels. “Randomized” indicates counts of AUC values obtained after randomly shuffling the sample labels

Flavour tags were also capable of characterizing commercial cultivars in terms of the given species tag. Figure 2 shows the network constructed using flavour similarity to weight the links between cultivars, e.g. the correlation between the *F*(*s*) vectors. The resulting network is shown in Fig. 2a Application of the Louvain algorithm yielded Q = 0.221 and a total of 19 modules, with the four largest containing ≈ 98% of all cultivars, see Supplementary information 5. In this case, modules composed predominantly of a single species tag were no longer clearly visible; however, a gradient of species tags (from “indicas” to “hybrids” to “sativas”) could be observed from top to bottom.

As we observed in the case of reported effects, flavours also showed that not only cultivars with similar naming conventions were grouped together, but also that their grouping was related to the flavours represented in their names (Fig. 2b). For instance, blueberries were grouped together and close to a cluster of grapes (I), fruit and cheese cultivars were in the same subpanel (II), fruit-related cultivars (pineapple, tangerine, citrus, orange) were grouped together (III), as well as skunks and diesels (IV), mangos and strawberries (V), with lemons appearing cohesively clustered together (VI). In this case, we must consider the possibility of bias due to the cultivar names in the reported flavour tags.

Interestingly, when using the flavour tag frequencies as features in a random forest classifier trained to distinguish “indicas” from “sativas”, we also obtained a highly accurate classification (Fig. 2c), with <AUC> = 0.828 ± 0.002 (mean ± STD, *p* < 0.001).

**Fig. 3:**
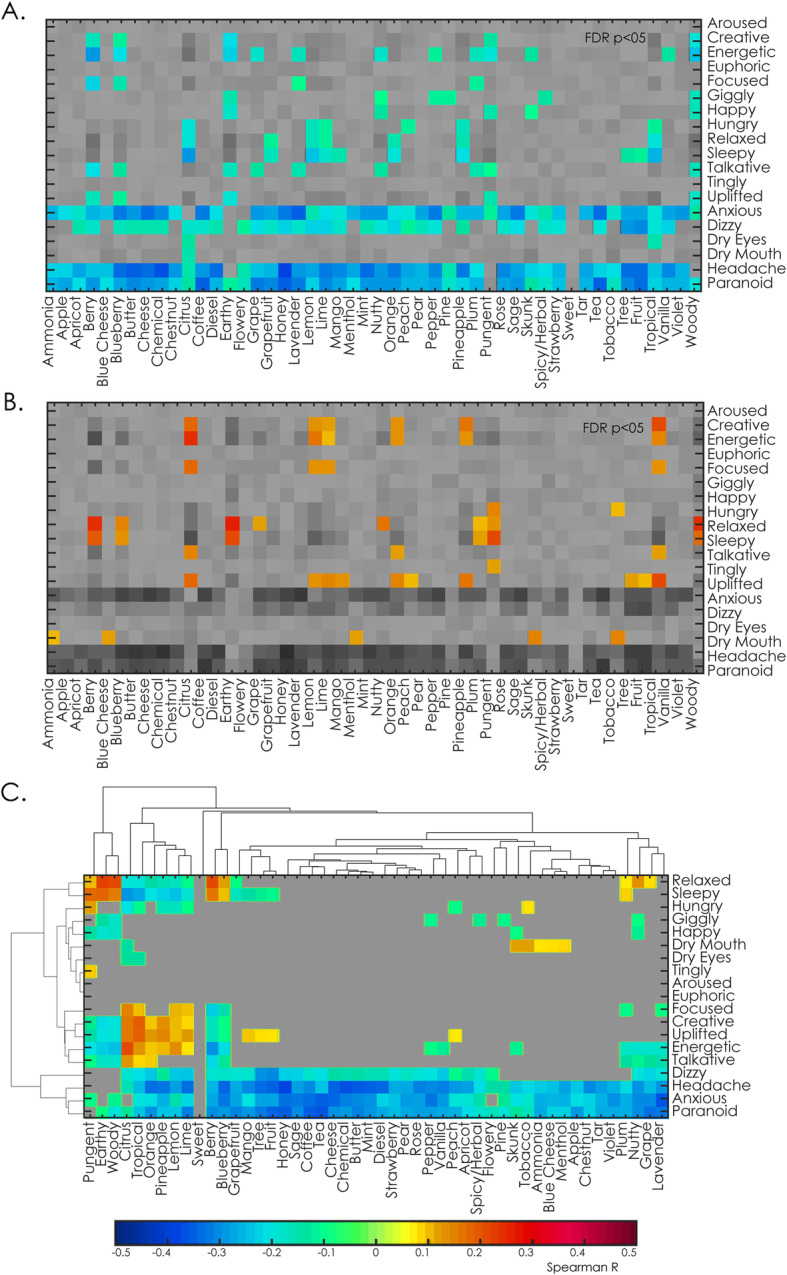
Associations between effects and flavours. A. Significant negative Spearman correlations between effects and flavours. B. Significant positive Spearman correlations between effects and flavours. In both panels significant correlations are indicated using a color scale. Both panels were thresholded at *p* < 0.05, FDR-corrected). C. Both positive (Panel A) and negative (Panel B) values grouped by hierarchical clustering of the effects and flavours according to their correlations. Hierarchical clustering was determined by the standard method included in Scipy (agglomerative clustering based on the Euclidean distance)

According to our next hypothesis, we evaluated the correlations between effect and flavour tags across cultivars, establishing a relationship between effects and flavour tags. The results are shown in Fig. 3. We found significant (*p* < 0.05, FDR-corrected) negative and positive effect-flavour correlations. Figure 3a shows negative correlations, i.e. inverse relationships between the frequency of the reported effect and flavour tags, while Fig. 3b illustrates positive correlations. The frequency of unpleasant subjective effects, such as “anxious”, “dizzy”, “headache” and “paranoid”, correlated negatively with the frequency of almost all flavour tags, meaning that users tended to avoid the use of flavour tags when describing unpleasant experiences. Complementary, we correlated cannabinoid content and reported effects for 183 “strains” that included cannabinoid content from PSI Labs and did not find an association between negative effects and THC content in this sample (see Supplementary Fig. 5). This could be explained by considering that negative subjective experiences may outweigh flavour or scent perception. This result also suggests that in these specific experiences the appreciation of aromatic and/or flavour variables is undermined by the overwhelming subjective effects. In these cases, flavors cannot explain unpleasant effects. Further inspection of Fig. 3a and b reveals that certain flavours, such as “berry”, “blueberry”, “earthy”, “pungent” and “woody”, were negatively correlated with subjective stimulant effects, such as “creative” and “energetic”, and at the same time presented positive correlations with soothing effects such as “relaxed” and “sleepy”. Other flavours, such as “citrus”, “lime”, “tar”, “nutty”, “pineapple” and “tropical” presented the opposite behaviour, i.e. they correlated negatively with soothing effects (“relaxed”, “sleepy”) and positively with stimulant effects (“creative”, “energetic”). The fact that the aforementioned flavours presented inverse correlation patterns with respect to opposite psychoactive effects adds support to the validity of this analysis.

Next, we performed a hierarchical clustering of the effects and flavours according to their correlations (Fig. 3c). The main cluster separated unwanted effects from the rest. The remaining clusters of subjective effects were divided into three categories: soothing (“relaxed”, “sleepy”), stimulant (“euphoric”, “creative”, “energetic”, “talkative”) and other miscellaneous effects commonly associated with cannabis use (“hungry”, “giggly”, “happy”, “dry mouth”, “dry eyes”, “tingly” and “aroused”). It is important to note that this hierarchy emerged from considering effect-flavour correlations only. Consistently, flavours were clustered according to their negative correlations (“pungent”, “earthy”, “woody”, “berry”, “blueberry”) and their positive correlations (“citrus”, “tropical”, “orange”, “pineapple”, “lemon”, “lime”).

**Fig. 4:**
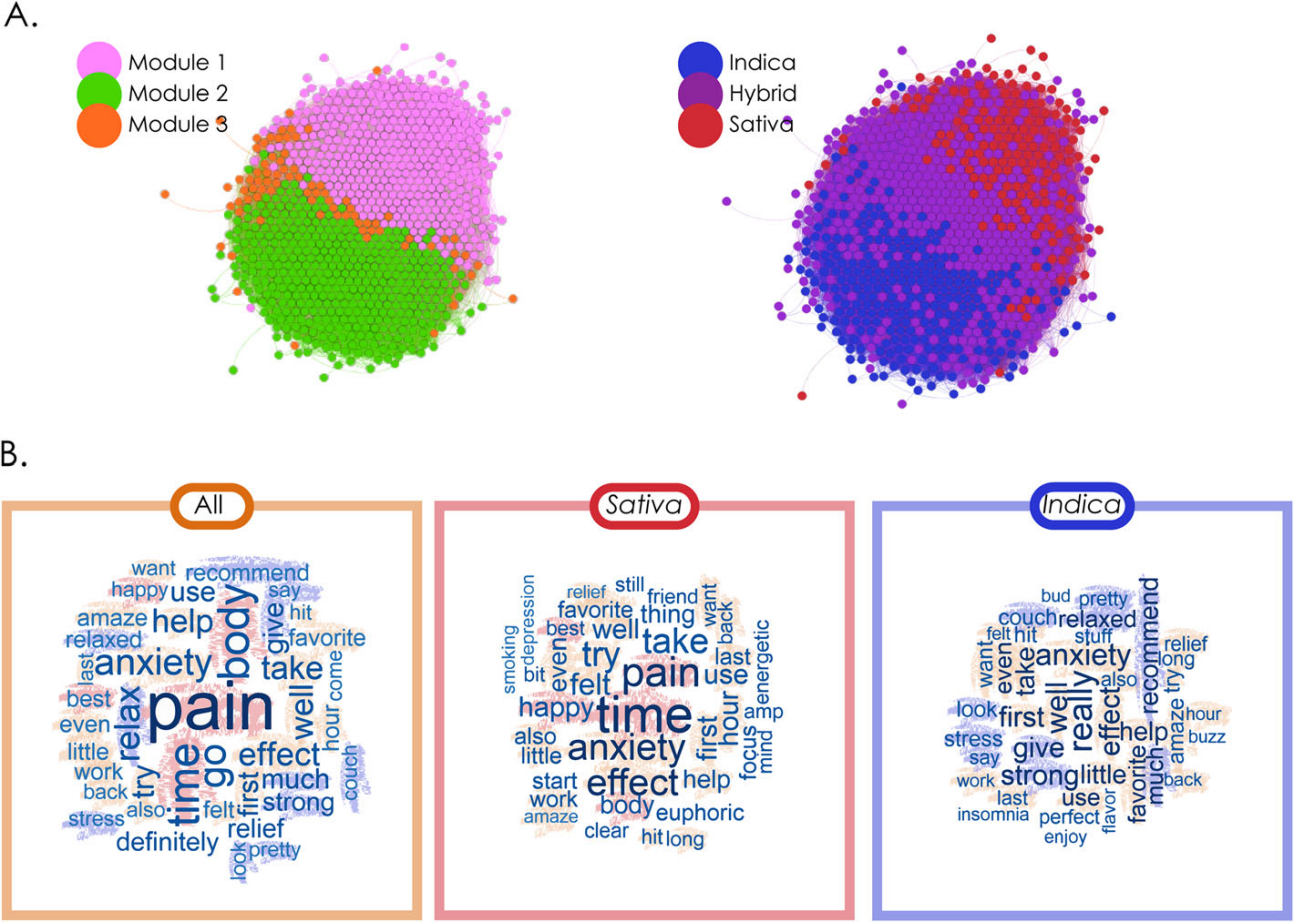
Analysis based on the semantic content of unstructured written reports. A. Networks constructed based on the semantic similarity of the reports associated with cultivars. The left panel is color-coded based on the results of modularity maximization using the Louvain algorithm, while the right panel is color-coded based on species tag. B. Word clouds representing the most frequent terms appearing in the reports of all cultivars combined (left), “sativas” (middle) and “indicas” (right)

Next, we tested our third hypothesis by objectively analysing the unstructured written reports with LSA and using this information to correlate cultivars and detect recurrent topics, which allowed us to relate the reports with the subjective effect tags. We found that the information contained in the self-reported tags was consistent with the free narratives provided by the users. Unstructured written reports can provide complementary information, since users are not limited by predefined sets of effect and flavour tags. We constructed a network in which nodes represented cultivars and links were weighted by their semantic similarity, measured by the correlation between the columns of the rank-reduced term-document matrix ${\hat{A}}_{50}$ (see the “Natural language processing of written unstructured reports” section in the Methods). The resulting networks are shown in Fig. 4a. Applying the Louvain algorithm yielded Q = 0.058, with a total of 15 modules, the largest 4 containing ≈ 98% of all cultivars, see Supplementary information 5. Module distribution was bimodal, i.e. when compared in terms of unstructured written reports, most cultivars fell into one of two categories. When comparing the modular decomposition with the species tag distribution, we found a clear division in terms of “indicas” and “sativas”, with “hybrids” in between. This division paralleled the two main modules. Module 1 was conformed predominantly by “sativas” and “hybrids”, while module 2 was conformed by “indicas” and “hybrids”.

Next, we investigated the most frequently used terms in the reports of all the cultivars taken together, and of “indicas” and “sativas” considered separately. Figure 4b presents word cloud representations of the 40 most common terms for cultivars. The most common terms related to the subjective perceptual and bodily effects (terms like “amaze”, “strong”, “felt”, “favourite”, “body”), therapeutic effects and/or medical conditions (“pain”, “anxiety”, “relax”, “help”, “relief”, “focus”) and emotions (“euphoric”, “anxiety”, “happy”, “confusion”). It is important to note that, due to limitations in the amount of available data, this analysis used single term representations (1-g), therefore words used in positive or negative contexts could not be differentiated, e.g. the term “anxiety” could appear in “This helped calm my anxiety” or in “This caused me anxiety” without distinction. Half of the most representative words were common to both “indicas” and “sativas”, such as “anxiety,” “amaze”, “effect”. The main difference between species tags emerged after excluding terms common to both, resulting in words such as “focus”, “euphoric”, “energetic” for sativas, and “insomnia”, “enjoy”, “flavour” for “indicas”. A detailed analysis of the main 5 components by species can be found in Supplementary Information 4 (see Supplementary Fig. 7).

**Fig. 5:**
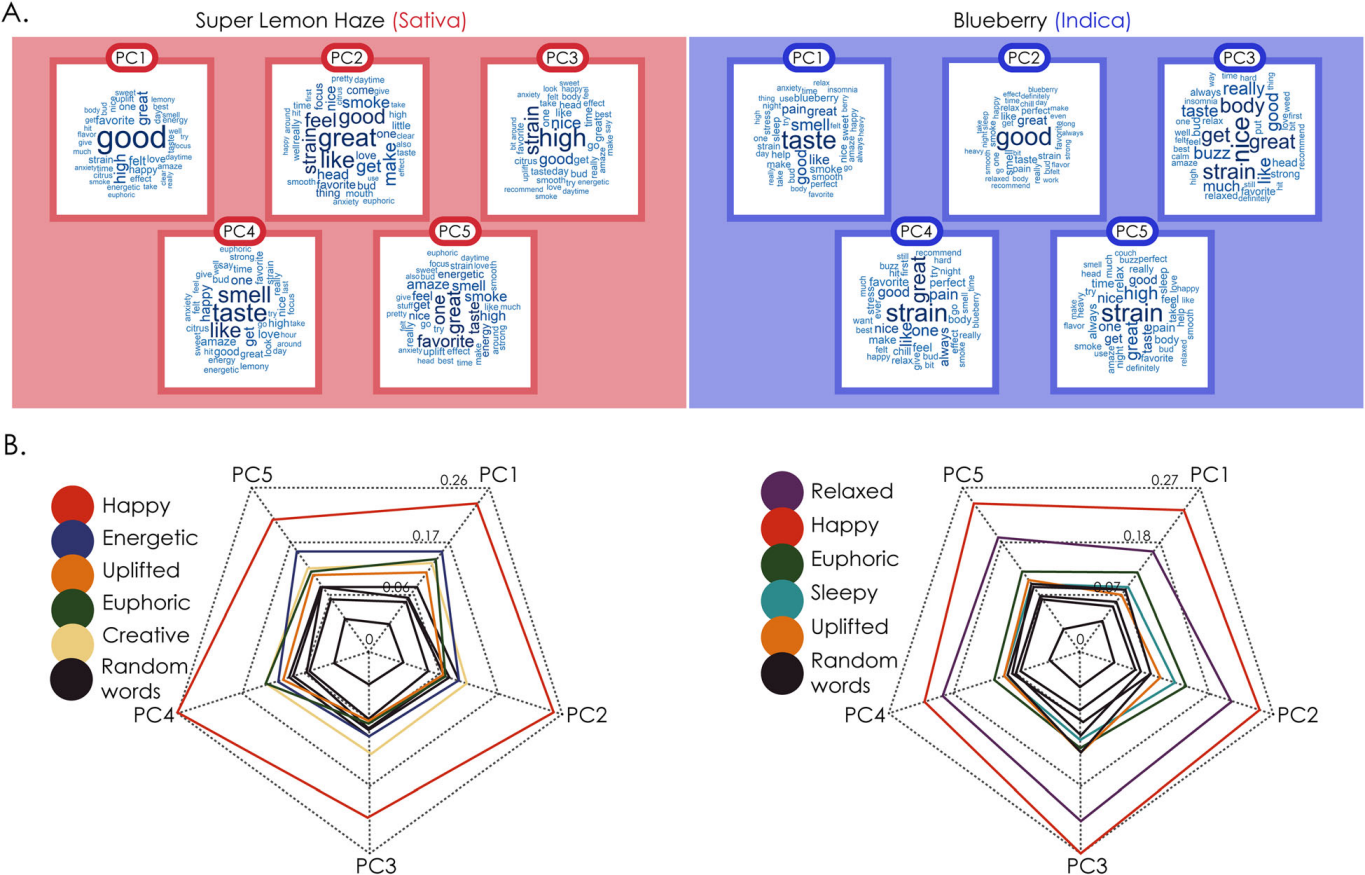
Correspondence between topics extracted from unstructured written reports and the choice of predefined tags. A. Word clouds of the first five principal components for the cultivars Super Lemon Haze and Blueberry, indicating the most representative topics within the associated reports. B. Radar plots showing the mean semantic similarity between the words in each topic and the most frequently chosen effect tags for both cultivars. It can be seen that the semantic similarity is the highest for the most frequently used tags, and the lowest for a set of randomly chosen words unrelated to the effects of cannabis

To relate the free narrative reports to the subjective effect tags, we investigated two cultivars with a large number of reports: Super Lemon Haze (“sativa”, *N* = 1.373, most frequently reported tags: “happy”, “energetic”, “uplifted”) and Blueberry (“indica”, *N* = 1456, most frequently reported tags: “relaxed”, “happy”, “euphoric”). We first applied PCA to the corresponding rank-reduced term-document frequency matrices to obtain the main topics for each “strain”. The word clouds with the highest-ranking terms for the first 5 principal components of each cultivar are presented in Fig. 5a. The variance explained by the first 5 components was 21% for Super Lemon Haze and also 21% for Blueberry. Next, we computed the semantic distance between the most frequent effect tags of each cultivar and the top 40 words in each of the principal components. The objective of this analysis was to evaluate whether the unstructured written reports reflected the choice of predefined tags made by the users. As shown in Fig. 5b, the most frequently reported effect tags for each cultivar showed a prominent projection into all the components, as compared to randomly chosen words. This suggests that users selected predefined tags consistently with the contents of their written reports.

### Terpene and cannabinoid content

Finally, in order to test our last hypothesis, we investigated the relationship between the user reports and the molecular composition of the cultivars. For this purpose, we accessed publicly available data of cannabinoid content provided in the work of Jikomes and Zoorob (Jikomes and Zoorob 2018), as well as assays of cannabinoid and terpene content from the PSI Labs website.

**Fig. 6:**
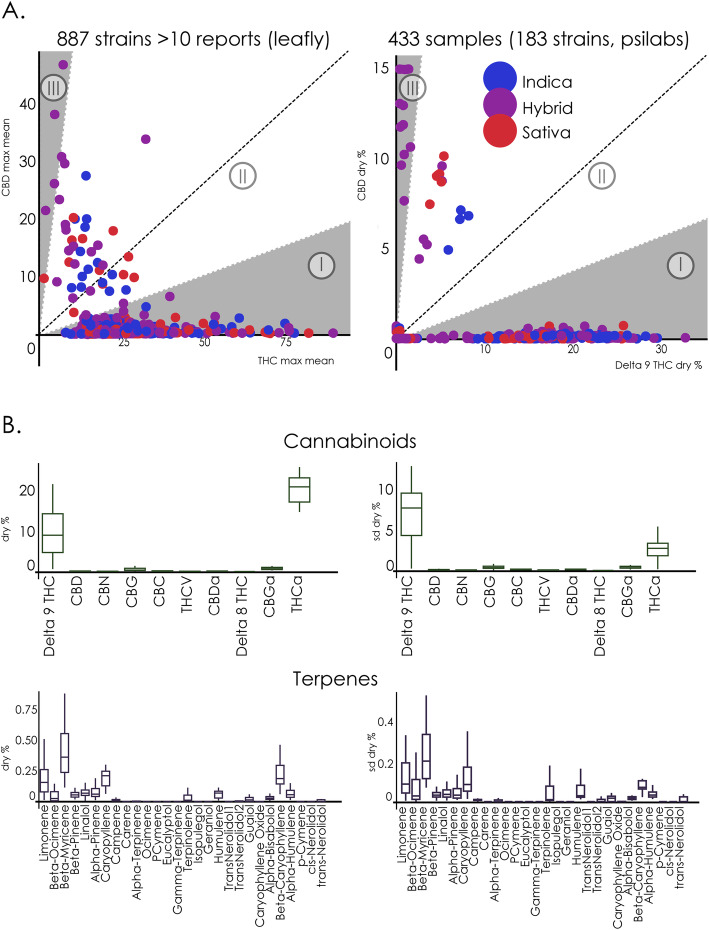
Chemical composition of cannabis cultivars. A. Scatter plot of CBD vs. THC (max/mean content) for all cultivars (left panel) and for the 183 cultivars included in the PSI Labs dataset, by dry % (right panel). The background is divided by chemotype (THC:CBD ratios), where Chemotype I indicates 5:1, Chemotype III indicates 1:5, and Chemotype II is in the middle of both (Jikomes and Zoorob 2018). While three different chemotypes could be identified, in both cases chemotype I (high THC content) was clearly overrepresented in the data. B. Cannabinoid and terpene content data extracted from PSI Labs. Left: boxplots of the mean dry content of 10 cannabinoids and 26 terpenes across multiple samples of the same strain. Right: the variability of the mean dry content across samples of the same cultivar (mean ± STD)

The first dataset contains information on THC and CBD content for all 887 cultivars studied in this work. The relationship between the content of both active cannabinoids is plotted in Fig. 6a, left panel. As reported by Jikomes and Zoorob, the cultivars fell into three general chemotypes based on their THC:CBD ratios (Jikomes and Zoorob 2018), consistent with previous findings (Hazekamp et al. 2016; Hillig and Mahlberg 2004; Jikomes and Zoorob 2018). Most of the investigated cultivars fell into chemotype I (Chemotype I: 94.6%, Chemotype II: 4.8%, Chemotype III: 0.6%), indicating high THC vs. CBD ratios. This was replicated using the cannabinoid content data obtained from PSI Labs (*N* = 433 individual flower samples corresponding to 183 different cultivars), as shown in Fig. 6a, right panel. Again, the majority of the assays corresponded to chemotype I (Chemotype I: 90.3%, Chemotype II: 6%, Chemotype III: 3.7%).

Figure 6b shows the compiled data for 10 cannabinoids and 26 terpenes across multiple samples of a cultivar included in the PSI Labs dataset. While some terpenes appeared to be robustly detected in the “strain”, the relatively large spread indicated a considerable level of variability.

**Fig. 7:**
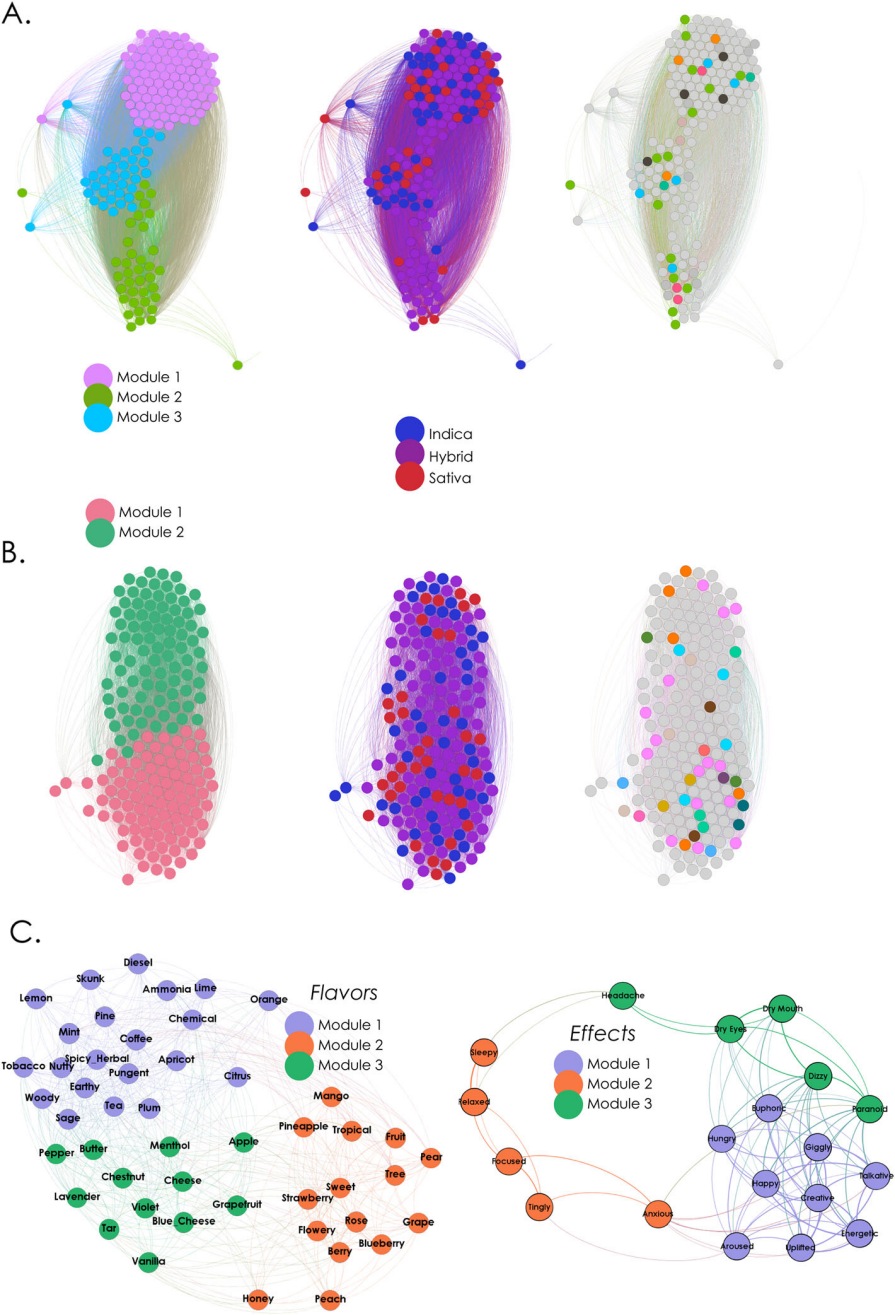
Association of cultivars, effect tags, and flavour tags in terms of chemical composition. A. Network of similarity in cannabinoid content. Each node represents a commercial “strain”, and their spatial proximity is based on the correlation between their corresponding cannabinoid profiles. Nodes in the left panel are color-coded based on modularity analysis, while nodes in the right panel are color-coded based on species tags. B. Same analysis as in Panel A, but for the similarity in terpene content. C. The network on the left represents the association between flavour tags, based on the correlation of the terpene profiles averaged across all cultivars for which the corresponding flavour tags were reported. The network in the right presents the same analysis for effect tags

Next, we addressed in more detail the association between cannabinoid content, terpene content, flavours, effects, and cannabis species tag. For this purpose, each of the 183 cultivars in the PSI Labs dataset was described by a cannabinoid and terpene vector. We computed the Spearman correlation between these vectors to weight the links connecting the nodes that represented the cultivars. This resulted in cannabinoid and terpene similarity networks, which are shown in Fig. 7a and b, respectively. The network on the left panel of Fig. 7a is color-coded based on the application of the Louvain algorithm (Q = 0.041) to the cannabinoid similarity network, yielding a total of 8 modules, with the largest 3 represening ≈ 94% of the cultivars. This modular structure paralleled the classification into the three chemotypes.

The network on the right is color-coded based on cannabis species tag: the first and largest module contained cultivars belonging to all species tags (similar to chemotype I); another module, situated in the middle, presented a more balanced proportion of species tags, but also contained a smaller proportion of cultivars (similar to chemotype II), and the remaining module was composed mostly by “hybrids” (as in chemotype III). Since this classification used more information than the THC:CBD ratios, it corresponds to a multi-dimensional analogue of the standard chemotype characterization.

Figure 7b shows the network obtained by correlating cultivars by their terpene vectors. The network on the left is color-coded based on the results of the Louvain algorithm (Q = 0.245), yielding only two modules. The network on the right is color-coded based on cannabis species tags. Since there are multiple terpenes in cannabis, without equivalents of main active cannabinoids such as THC and CBD, the chemical description in terms of terpenes is necessarily multi-dimensional. As with the semantic analysis of written reports, the association of cultivars based on the terpene profiles was bimodal and without a clear differentiation in terms of species tags.

Finally, we explored how effects and flavours were related based on the terpene content of the cultivars (Fig. 7c). We generated a terpene vector associated with each effect and flavour tag by averaging the terpene content across all the cultivars for which that tag was reported. The left panel of Fig. 7c shows how flavour tags (nodes) relate in terms of the correlation of their associated terpene vectors (weighted links). Modularity analysis (Q = 0.324) yielded a module comprising intense and pungent flavours (“skunk”, “diesel”, “chemical”, “pungent”) combined with citric flavours (“lemon”, “orange”, “lime”, “citrus), a second module containing sweet and fruity flavours (“mango”, “strawberry”, “sweet”, “fruit”, “grape”), and a third module with a mixture of salty and sweet flavours (“cheese”, “butter”, “vanilla”, “pepper”), see Supplementary information 5. Modularity analysis (Q = 0.194) of the network of effect tags associated by terpene similarity (Fig. 7c, right panel) yielded three modules resembling the clustering of effects presented in Fig. 3c, where we found groups consisting of subjective unwanted effects, stimulant effects and soothing effects, with an intermediate group associated with miscellaneous effects of smoked cannabis. Module 1 contained mostly stimulant effects (“energetic”, “euphoric”, “creative”, “talkative”, among others), module 2 contained soothing effects (“sleepy”, “relaxed”), and module 3 contained unwanted effects such as “headache”, “dizzy”, “paranoid” (with the exception of “anxious”, which was included in module 2). The fact that the network of effects associated by terpene content similarity reflected the hierarchical clustering of effects obtained from flavour association (Fig. 3c) reinforces the link between flavours and the psychoactive effects of cannabis.

## Discussion

We presented a novel synthesis of multi-dimensional data on a large number of cannabis cultivars with the purpose of developing an integrated view of the relationship between reported subjective effects, perceptual profiles (flavours) and chemical composition (terpene and cannabinoid content). As a result of this analysis, we established that cannabis species tags can be inferred from self-reported effect and flavour tags (Figs. 1 and 2, panel C), as well as from unstructured written reports, which also revealed that topics associated with subjective effects had different prevalence in “indicas” compared to “sativas”. This classification was obtained using supervised (random forests) and unsupervised (network modularity maximization) methods, confirming our first hypothesis, which stated that these methods would group similar cultivars together. As suggested by the previous literature (Casano et al. 2011; Fischedick and E. S. 2015; Pollastro et al. 2018), we found that classifiers based on the reported flavours achieved high accuracy in the classification of commercial cultivars. Furthermore, flavour and effect tags did not manifest independently, but presented significant correlations (Russo 2011, 2019), which we expected in our original hypothesis. Finally, in spite of high variability in the reported chemical compositions, we corroborated the presence of expected flavour-terpene associations, validating our last hypothesis. In the following, we discuss the implications of our work in the context of leveraging large volumes of data produced under naturalistic conditions in combination with quantitative chemical analyses for the classification and characterization of commercial cannabis cultivars.

The practical relevance of our results is manifest in the need to develop fast, cheap and reliable methods for cannabis cultivar characterization. We found that available crowdsourced data was useful to recognize species (Figs. 1 and 2, panel C). Over the past years, cannabis plant species (“indica” / “sativa”) have been challenged by the scientific community as reliable markers of the effects elicited by the consumption of the plant (Piomelli and Russo 2016; Pollastro et al. 2018; Russo 2019), pointing towards objective chemotype descriptors (mainly THC:CBD ratios) as a new gold standard. According to this characterization, THC is often considered the active compound related to many of the negative outcomes of cannabis consumption (Volkow et al. 2016), while CBD (or combinations of CBD and THC) is associated with most of the purported medicinal properties (Fogaça et al. 2018; Hahn 2018; Hurd et al. 2019; Lorenzetti et al. 2016; Nadulski et al. 2005; Nuutinen 2018; Russo 2019; Vandrey et al. 2015). Although there is no doubt that a precise chemical description of the plant is the most accurate and reliable predictor of the elicited subjective effects, this approach is likely impractical in the present market (Nie et al. 2019). In the first place, this approach requires technology for quantitative chemical analysis that is beyond the reach of many dispensaries and growers. Furthermore, variations in the concentration of cannabinoids are high even within the same “strain”, depending on factors such as age, environmental conditions, generation, and geographical location (Casano et al. 2011; Fetterman et al. 1971; Nuutinen 2018; Russo 2011). Finally, the predictive value of chemotypes has been questioned in markets where consumers increasingly demand higher THC content (Freeman et al. 2019, Freeman et al. 2018; Jikomes and Zoorob 2018; Smart et al. 2017).

Our results suggest that perceptual profiles (reported flavours) and terpene quantification show merit for the characterization of cannabis cultivars. Both tagged subjective effects and perceived flavours were capable of explaining the Leafly species tags given by the users with high accuracy (Figs. 1 and 2, panel C). It should be noted that the word “explain” used in this context refers to the estimation of the class of a sample given the fitted model (Shmueli 2010). Concerning the interpretation of the machine learning classification, we see two possible reasons for the significant classification into “indicas”, “sativas” and “hybrids”. First, the classifications made by the users might not be entirely arbitrary and could be based on botanical (or other) characteristics that are suggestive of different classes of breeds. Second, there could be biases not related to the breeds per se that drive the associations made by the users. In either case, even if the tags do not reflect a proper botanical classification, we consider it important that the assessments of the breeds made by the users are clustered, and that these clusters reflect classifications that (even if outdated in botanical terms) are still relevant to label cannabis in a commercial context. Although it has been recently proposed by some authors to eliminate this species classification regarding cannabis plant (Piomelli and Russo 2016) our results may support that some of the underlying differences amongst these categories (Lamarck 1785) were conserved over time, in agreement with the results obtained by Hilling and Mahlberg (Hillig 2004; Hillig and Mahlberg 2004); even though more detailed chemical characterizations should be obtained to assess this speculation. Terpenes are highly conserved across generations (Aizpurua-Olaizola et al. 2016; Casano et al. 2011), can be synergistic with cannabinoids (Russo 2011), and have psychoactive properties by themselves (Nuutinen 2018). It follows from our analysis that users likely count on perceptual faculties to select cultivars when seeking specific effects. Further research in controlled laboratory settings is required to test the capacity for assessing psychoactive effects based on sensory information. Moreover, the reported flavour-effect correlation could provide users valuable information concerning the expected effects based on the aroma of the plant. In particular, Fig. 3 shows the dendrogram structure for flavors and effects. Flavours fell into four broad categories: earthy, citrics, berries, and others. Effects were clustered into three main categories, negative, uplifted and relaxed. The correlation analysis provided information concerning the interaction between these two classifications. This result is consistent with the previous rich literature on flavour-effect relationships (Blank et al. 2011; Delwiche 2004; Holland and Gallagher 2004; Levin et al. 1990; Small and Prescott 2005) and suggests a possible modulating effect of flavours on subjective effects.

There is increasing evidence that the subjective effects of cannabis are a result of the synergy between a diverse group of active ingredients which include THC and CBD, alongside other cannabinoids and terpenes (Baron 2018; Nuutinen 2018; Russo 2011). This observation supports the need for a multi-dimensional characterization that does not neglect terpene content, and therefore the associated flavours. We found that, even with overall high levels of THC across all cultivars (Jikomes and Zoorob 2018), the subjective experiences reported by users were capable of clustering cultivars by species tags, not only based on effects but also on the reported flavours. Moreover, the clustering of cultivars with names evoking certain flavours, even while not botanically validated (Clarke and Merlin 2013), supported that terpene content is a well-preserved property in the cultivars.

As in the “sativa”-“indica” gradient revealed by the analysis of effect and flavour tags, the semantic analysis of unstructured written reports clearly captured the distinction between “sativa like” and “indica like” cultivars. It is interesting to note that, in spite of overall high THC content across all cultivars, the specific words that emerged from LSA topic detection applied to reports of “sativas” and “indicas” represented a large proportion of positive and desired effects, such as relaxing and uplifting effects (Corral 2001). This is consistent with the relatively low value of Q for the LSA network among all cultivars (see Supplementary information 5). Natural language analysis also established that, even while therapeutic and subjective effects were thoroughly discussed throughout the written reports, seed acquisition and plant growing were also prominently featured.

Concerning terpene and cannabinoid profiles, it is important to note that cannabinoids showed a clear trimodal structure, in accordance with the three chemotypes described by Jikomes & Zoorob (Jikomes and Zoorob 2018), which were obtained based only on THC and CBD concentrations. The subjective effect tags could be clustered into three main groups, congruent with those obtained from the Leafly flavour tags: Soothing, Stimulant, Unpleasant (Fig. 3 and Fig. 7, c). This led us to believe that similar behavior would be observed when reporting negative or unwanted effects (which apply arbitrarily to plants with different terpene profiles). This suggests that factors independent of plant characteristics (e.g. set and setting) are the principal cause of negative experiences, as already suggested by previous works (Hartogsohn 2016; McElrath and McEvoy 2002). Moreover, even though the modules were stable, the Q values were low, which is consistent with the large variability of this dataset (see Supplementary information 5). The fact that a trimodal grouping of the cultivars was also obtained based on 10 cannabinoids could imply that the complex interactions of a larger number of active molecules might project into a reduced tri-dimensional space without significant loss of information. The concept of multi-dimensional chemotype should be further explored in controlled laboratory conditions to develop more accurate objective descriptors of different cannabis cultivars and their elicited effects. Conversely, cultivars were organized bimodally by their terpene content. This observation is interesting in the context of the flavour-effect associations identified in our work, which were essentially organized into two groups: stimulating and sedative effects. These associations add support to the active role of terpenes (Nuutinen 2018). The analysis of effect association via terpene content similarity yielded results convergent with those obtained from correlating flavour and effect tags, adding further support to the suggestion that psychoactive effects could by mediated by terpenes.

The strengths of our study stem from the analysis of large volumes of data impossible to obtain under controlled laboratory conditions, but this approach also leads to limitations inherent to self-reporting by users, preventing objective verification of the consumed cultivars, as well as their doses or whether the cultivar names biased the perceptual reports. To avoid bias given by “strain” names, the optimal solution could be a blind rating for all “strains”. While this data is not available at the moment, it could be valuable in the future. To partially address this limitation, we carefully analyzed our sample, ensuring that the maximum possible number of non-independent samples was one order of magnitude minor than the size of our dataset, and that 5-folds cross validation was always used to maintain an independent sample for model evaluation. The possibility of a certain measure of dependence within our dataset remains, which should be considered as a limitation of our analysis. However, commercial strains with similar flavors were clustered together by their terpene content, suggesting that “strain” names are driven by the chemical composition of the plants. Given that consumption of commercial cannabis might carry expectations (e.g. related to cultivar names, reviews, or past experiences by the user), we stress that our results could be influenced by these and other contextual variables, which is usually the case in large-scale studies of psychoactive drugs in naturalistic settings (Carhart-Harris et al. 2018; Olson et al. 2020). Conducting this kind of research following placebo-controlled designs is impossible, therefore our results are valuable yet only within the boundaries imposed by these limitations. Concerning the methodology, even though the Louvain algorithm is one of the most popular clustering methods used in the bibliography, recently there has been evidence that this method could present certain issues regarding community identification (Traag et al. 2019). This should be taken into account when drawing conclusions from the clustering of the data. Limitations are also inherent to the assumption of cannabis cultivars as having homogeneous chemical compositions. Previous work showed that cannabinoid content can present ample variation within single cultivars (Fischedick and E. S. 2015; Jikomes and Zoorob 2018), and our results show that similar considerations apply to terpene profiles. Given our results, we can hypothesize that large-scale chemical screening of cannabinoid content and terpenes should reveal systematic associations between both variables, and that these associations should parallel those here presented. Moreover, it has long been recognized that flavour profiles interact with pleasurable subjective effects in other drugs, such as the case of tobacco smoked in cigarettes (Blank et al. 2011; Levin et al. 1990), with flavor-effect interactions being broadly recognized and studied in terms of their neurophysiological basis (Delwiche 2004; Small and Prescott 2005) and studied on this neurophysiological basis (Holland and Gallagher 2004). Our results suggest similar interactions in the case of cannabis: besides the main psychoactive compounds (cannabinoids), flavours and odours (depending on terpenes and flavonoids) can exert, modulate and/or interact with the subjective effects elicited by cannabis. Future studies could address a smaller sample of cultivars more thoroughly investigated in terms of their chemical composition, thus allowing the study of correlations between self-reported subjective effects, flavours, and environmental factors that could impact on cannabinoid and terpene content.

## Conclusions

After decades of prohibition, the legal cannabis industry for therapeutic and recreational use is growing at a quick pace, but nevertheless it is at its infancy. Considerable evidence suggests that commercially available cultivars are highly variable in their chemical composition and subjective effects. In comparison, more mature industries, such as that of winery, have arrived at reliable standards (e.g. Merlot, Cabernet, Syrah) that are trusted and understood by the consumers. By extracting information from different sources of data, our work suggests that the development of standards in the cannabis industry should not only focus on psychoactive effects and cannabinoid content, but also take into account scents and flavours, which constitute the perceptual counterpart of terpene and terpenoid profiles.

## Supplementary information

**Additional file 1.** Supplementary tables with details on cannabis cultivars, flavour and effect tags, and supplementary analyses to support the results.

## Data Availability

The datasets supporting the conclusions of this article are available in the Mendeley Data repository (DOI: 10.17632/6zwcgrttkp.1, https://tinyurl.com/yyzkk78r).

## Abbreviations

THC: Delta-9-Tetrahydrocannabinol
CBD: Cannabidiol
STD: Standard deviation
AUC: Area under the receiver operating characteristic curve
LSA: Latent Semantic Analysis
SVD: Singular Value Decomposition
PCA: Principal Component Analysis
FDR: False Discovery Rate

## References

[CR1] Adams JL (2019). CALIFORNIA SALES TAXES ON BUSINESS SERVICES.

[CR2] Aizpurua-Olaizola O, Soydaner U, Öztürk E, Schibano D, Simsir Y, Navarro P (2016). Evolution of the cannabinoid and Terpene content during the growth of Cannabis sativa plants from different Chemotypes. J Nat Prod.

[CR3] Baron EP (2018). Medicinal properties of cannabinoids, Terpenes, and flavonoids in Cannabis, and benefits in migraine, headache, and pain: an update on current evidence and Cannabis science. Headache.

[CR4] Bastian M, Heymann S, Jacomy M (2009). Gephi: an open source software for exploring and manipulating networks. Proceedings of International AAAI Conference on Web and Social Media.

[CR5] Blank MD, Cobb CO, Kilgalen B, Austin J, Weaver MF, Shihadeh A, Eissenberg T (2011). Acute effects of waterpipe tobacco smoking: a double-blind, placebo-control study. Drug Alcohol Depend.

[CR6] Blondel, V. D., Guillaume, J. L., Lambiotte, R., & Lefebvre, E. (2008). Fast unfolding of communities in large networks. Journal of Statistical Mechanics*:* Theory and Experiment. 10.1088/1742-5468/2008/10/P10008.

[CR7] Bonini SA, Premoli M, Tambaro S, Kumar A, Maccarinelli G, Memo M, Mastinu A (2018). Cannabis sativa: a comprehensive ethnopharmacological review of a medicinal plant with a long history. J Ethnopharmacol.

[CR8] Carhart-Harris RL, Roseman L, Haijen E, Erritzoe D, Watts R, Branchi I, Kaelen M (2018). Psychedelics and the essential importance of context. J Psychopharmacol.

[CR9] Casano, S., Grassi, G., Martini, V., & Michelozzi, M. (2011). Variations in Terpene profiles of different strains of Cannabis sativa L. CRA-CIN Consiglio per la Ricerca e la Sperimentazione in Agricoltura Centro di Ricerca per le Colture Industriali Rovigo Italy, 115–122.

[CR10] Clarke, R. C., & Merlin, M. D. (2013). Cannabis: EVOLUTION AND ETHNOBOTANY. UNIVERSITY OF CALIFORNIA PRESS.

[CR11] Corral VL (2001). Differential effects of medical marijuana based on strain and route of administration. J Cannabis Therapeutics.

[CR12] Coulomb (1785). Premier mémoire sur l’électricité et le magnétisme. Histoire de l’Académie Royale des Sciences.

[CR13] De Meijer EPM, Hammond KM, Sutton A (2009). The inheritance of chemical phenotype in Cannabis sativa L. (IV): cannabinoid-free plants. Euphytica.

[CR14] DeCarlo LT (1997). On the meaning and use of kurtosis. Psychol Methods.

[CR15] Delwiche, J. (2004). The impact of perceptual interactions on perceived flavor. Food Qual Prefer, 15(2), 137–146. 10.1016/S0950-3293(03)00041-7.

[CR16] Ermagun A, Punel A, Stathopoulos A (2020). Shipment status prediction in online crowd-sourced shipping platforms. Sustain Cities Soc.

[CR17] Fetterman PS, Keith ES, Waller CW, Guerrero O, Doorenbos NJ, Quimby MW (1971). Mississippi-grown cannabis sativa L.: preliminary observation on chemical definition of phenotype and variations in tetrahydrocannabinol content versus age, sex, and plant part. J Pharm Sci.

[CR18] Fischedick J, E. S. Cannabinoids and Terpenes as chemotaxonomic markers in Cannabis. Natural Products Chem Res. 2015;03(04) 10.4172/2329-6836.1000181.

[CR19] Fogaça MV, Campos AC, Coelho LD, Duman RS, Guimarães FS (2018). The anxiolytic effects of cannabidiol in chronically stressed mice are mediated by the endocannabinoid system: role of neurogenesis and dendritic remodeling. Neuropharmacology.

[CR20] Freeman TP, Groshkova T, Cunningham A, Sedefov R, Griffiths P, Lynskey MT (2019). Increasing potency and price of cannabis in Europe, 2006–16. Addiction.

[CR21] Freeman TP, Lynskey MT, Das RK, Van Der Pol P, Rigter S, Van Laar M (2018). Changes in cannabis potency and first-time admissions to drug treatment: a 16-year study in the Netherlands. Psychol Med.

[CR22] Gilbert AN, DiVerdi JA (2018). Consumer perceptions of strain differences in Cannabis aroma. PLoS One.

[CR23] Hahn B (2018). The potential of Cannabidiol treatment for Cannabis users with recent-onset psychosis. Schizophr Bull.

[CR24] Hartogsohn I (2016). Set and setting, psychedelics and the placebo response: an extra-pharmacological perspective on psychopharmacology. J Psychopharmacol.

[CR25] Hastie TT (2009). The elements of statistical learning. Math Intell.

[CR26] Hazekamp A, Tejkalová K, Papadimitriou S (2016). Cannabis: from cultivar to Chemovar II—A metabolomics approach to Cannabis classification. Cannabis Cannabinoid Research.

[CR27] Henry P. Cannabis chemovar classification: terpenes hyper-classes and targeted genetic markers for accurate discrimination of flavours and effects. PeerJ Preprints. 2017; 10.7287/peerj.preprints.3307.

[CR28] Hillig KW (2004). A chemotaxonomic analysis of terpenoid variation in Cannabis. Biochem Syst Ecol.

[CR29] Hillig KW, Mahlberg PG (2004). A chemotaxonomic analysis of cannabinoid variation in Cannabis (Cannabaceae). Am J Bot.

[CR30] Holland PC, Gallagher M (2004). Amygdala-frontal interactions and reward expectancy. Curr Opin Neurobiol.

[CR31] Hooke, R. (1678). Lectures de potentia restitutiva, or*, Of spring*.

[CR32] Huang TS, Narendra PM. Image restoration by singular value decomposition. Appl Opt. 2008; 10.1364/ao.14.002213. 10.1364/AO.14.002213 20154987

[CR33] Hurd, Y. L., Spriggs, S., Alishayev, J., Winkel, G., Gurgov, K., Kudrich, C., … Salsitz, E. (2019). Cannabidiol for the reduction of Cue-induced craving and anxiety in drug-abstinent individuals with heroin use disorder: a double-blind randomized placebo-controlled trial. American Journal of Psychiatry, appi.Ajp.2019.1. 10.1176/appi.ajp.2019.18101191. 10.1176/appi.ajp.2019.18101191 31109198

[CR34] James G, Witten D, Hastie T, Tibshirani R (2013). An introduction to statistical learning. Synthesis lectures on mathematics and statistics (Vol. 103).

[CR35] Jikomes N, Zoorob M (2018). The cannabinoid content of legal Cannabis in Washington state varies systematically across testing facilities and popular consumer products. Sci Rep.

[CR36] Kamalov F (2019). Sensitivity analysis for feature selection. Proceedings - 17th IEEE international conference on machine learning and applications, ICMLA 2018.

[CR37] Lamarck J (1785). Encyclopédique méthodique, Botanique I (part 2): 694–695.

[CR38] Landauer TK, Foltz PW, Laham D (1998). An introduction to latent semantic analysis. Discourse Process.

[CR39] Levin, E. D., Behm, F., & Rose, J. E. (1990). The use of flavor in cigarette substitutes. Drug Alcohol Depend, 26(2), 155–160. 10.1016/0376-8716(90)90122-U. 10.1016/0376-8716(90)90122-u 2242716

[CR40] Lorenzetti V, Solowij N, Yücel M (2016). The role of cannabinoids in Neuroanatomic alterations in Cannabis users. Biol Psychiatry.

[CR41] Martial C, Cassol H, Charland-Verville V, Pallavicini C, Sanz C, Zamberlan F (2019). Neurochemical models of near-death experiences: a large-scale study based on the semantic similarity of written reports. Conscious Cogn.

[CR42] McElrath K, McEvoy K (2002). Negative experiences on ecstasy: the role of drug, set and setting. J Psychoactive Drugs.

[CR43] Mechoulam, R. (2019). The Pharmacohistory of Cannabis sativa. In Cannabinoids as Therapeutic Agents 10.1201/9780429260667-1.

[CR44] Minarro-Gimenez, J. A., Marin-Alonso, O., & Samwald, M. (2014). Exploring the application of deep learning techniques on medical text corpora. In Studies in Health Technology and Informatics 10.3233/978-1-61499-432-9-584. 25160253

[CR45] Nadulski T, Pragst F, Weinberg G, Roser P, Schnelle M, Fronk EM, Stadelmann AM (2005). Randomized, double-blind, placebo-controlled study about the effects of cannabidiol (CBD) on the pharmacokinetics of Δ9-tetrahydrocannabinol (THC) after oral application of THC verses standardized cannabis extract. Ther Drug Monit.

[CR46] Nie B, Henion J, Ryona I (2019). The role of mass spectrometry in the Cannabis industry. J Am Soc Mass Spectrom.

[CR47] Nuutinen T (2018). Medicinal properties of terpenes found in Cannabis sativa and Humulus lupulus. Eur J Med Chem.

[CR48] Olson JA, Suissa-Rocheleau L, Lifshitz M, Raz A, Veissière SPL. Tripping on nothing: placebo psychedelics and contextual factors. Psychopharmacology. 2020; 10.1007/s00213-020-05464-5. 10.1007/s00213-020-05464-5 32144438

[CR49] Permanente, K., & Care, M. (2008). Journal of Cannabis Marijuana Use in HIV-Positive and AIDS Patients, 9775(October 2014), 37–41. 10.1300/J175v01n03.

[CR50] Piomelli D, Russo EB (2016). The Cannabis sativa versus Cannabis indica debate: an interview with Ethan Russo, MD. Cannabis Cannabinoid Res.

[CR51] Pollastro F, Minassi A, Fresu LG (2018). Cannabis Phenolics and their bioactivities. Curr Med Chem.

[CR52] Robinson, M. (2017). The legal weed market is growing as fast as broadband internet in the 2000s. Retrieved from https://www.businessinsider.com/arcview-north-america-marijuana-industry-revenue-2016-2017-1. Accessed Aug 2018.

[CR53] Russo EB (2011). Taming THC: potential cannabis synergy and phytocannabinoid-terpenoid entourage effects. Br J Pharmacol.

[CR54] Russo EB (2019). The case for the entourage effect and conventional breeding of clinical cannabis: No “Strain,” no gain. Front Plant Sci.

[CR55] Russo EB, Jiang HE, Li X, Sutton A, Carboni A, Del Bianco F (2008). Phytochemical and genetic analyses of ancient cannabis from Central Asia. J Exp Bot.

[CR56] Sanz, C., & Tagliazucchi, E. (2018). The experience elicited by hallucinogens presents the highest similarity to dreaming within a large database of psychoactive substance reports. Front Neurosci, 12(JAN), 1–19. 10.3389/fnins.2018.00007. 10.3389/fnins.2018.00007 PMC5786560 29403350

[CR57] Shmueli G (2010). To explain or to predict?. Stat Sci.

[CR58] Small DM, Prescott J (2005). Odor/taste integration and the perception of flavor. Exp Brain Res.

[CR59] Smart R, Caulkins JP, Kilmer B, Davenport S, Midgette G (2017). Variation in cannabis potency and prices in a newly legal market: evidence from 30 million cannabis sales in Washington state. Addiction.

[CR60] Traag VA, Waltman L, van Eck NJ (2019). From Louvain to Leiden: guaranteeing well-connected communities. Sci Rep.

[CR61] UNODC. (1968). A combined spectrophotometric differentiation of samples of cannabis. Retrieved from http://www.unodc.org/unodc/en/data-and-analysis/bulletin/bulletin_1968-01-01_3_page005.html. Accessed Aug 2018.

[CR62] Vandrey R, Raber JC, Raber ME, Douglass B, Miller C, Bonn-Miller MO (2015). Cannabinoid dose and label accuracy in edible medical cannabis products. JAMA.

[CR63] Volkow ND, Swanson JM, Evins AE, DeLisi LE, Meier MH, Gonzalez R (2016). Effects of Cannabis use on human behavior, including cognition, motivation, and psychosis: a review. JAMA Psychiatry.

[CR64] Weedmaps. (n.d.). Products and how to consume. Retrieved from https://weedmaps.com/learn/products-and-how-to-consume/edibles/. Accessed 2018.

[CR65] Zamberlan F, Sanz C, Martinez Vivot R, Pallavicini C, Erowid F, Erowid E, Tagliazucchi E (2018). The varieties of the psychedelic experience: a preliminary study of the association between the reported subjective effects and the binding affinity profiles of substituted phenethylamines and tryptamines. Front Integr Neurosci.

