# Supplementary material for: Relationship among subjective responses, flavor, and chemical composition across more than 800 commercial cannabis varieties
Source: J Cannabis Res. 2020 Jul 17;2:21. doi: 10.1186/s42238-020-00028-y (PMC7819481; doi:10.1186/s42238-020-00028-y)
Supplement: Supplementary file 1 — Additional file 1. Supplementary tables with details on cannabis cultivars, flavour and effect tags, and supplementary analyses to support the results. [file 42238_2020_28_MOESM1_ESM.pdf]

Relationship among subjective responses, flavor, and chemical composition across more than 800 commercial cannabis varieties

Supplementary data

1. Data sets ... 2

Leafly.com user's database by August 2018 ... 2

Supplementary Figure 1: Reports per subject per cultivar ... 2

Supplementary Figure 2: Example report by one cultivar. ... 3

Supplementary Table 1: Strains ... 4

Supplementary Table 2: Effects ... 10

Supplementary Table 3: Flavours ... 11

2. Methodology ... 11

Random Forest ... 12

Latent Semantic Analysis ... 13

Word2vec ... 13

Supplementary Figure 3: Outline of the methodology ... 14

3. Flavors and Effects ... 14

Supplementary Figure 4: THC and CBD relation with effects ... 14

Supplementary Figure 5: Flavors and Effects graph ... 16

Random Forest feature importance ... 16

4. Topics and LSA analysis ... 17

Supplementary Figure 6: LSA validation ... 17

Supplementary Figure 7: Topics ... 17

5. Graphs community stability ... 18

Supplementary Table 5 Modular stability ... 18

6. Bibliography ... 19

1. Data sets

It is controversial to use the term “strain” to refer to commercially available cannabis varieties. Some authors prefer to use it (Gilbert and DiVerdi, 2018) and others explicitly discuss and explore the predictive value of strain names (Jikomes and Zoorob, 2018). Lewis and Russo explored the chemical coherence among individual samples of different varieties and defined the chemovar as the preferred nomenclature (Lewis et al., 2018). Given that the chemovar could not be applied to the Leafly data (since we did not have the THC:CBD content of individual samples), we substituted the term “strain” for “commercial varieties” or “cultivars”, following (Lewis et al., 2018).

Concerning the species label, in spite of the recent proposal to eliminate this classification (Piomelli and Russo, 2016), historically, cannabis species have been reported to present different botanical characteristics, such as height, leaf width, etc (Bonini et al., 2018; LAMARCK, 1785), moreover, some contemporary authors have reported chemical characteristics specific to each particular species (Hillig, 2004; Hillig and Mahlberg, 2004). Although we do not state that this is the case for commercially available cultivars, we propose that some of the underlying differences between these categories could be conserved over time. Since we do not have any particular preference with respect to the category names, we adopted the “indica” and “sativa” labels because they were the ones provided by the Leafly website as well as by its users.”

Leafly.com user’s database by August 2018

The total number of reports by August 2018 was N reports = 100.901, with N = 983 corresponding to anonymous users. The remaining 99.918 reports corresponded to N users = 43.925, and 57.4% of these users completed only one report. Table 1 details the percentage of users with more than one, two and three reports. The last two columns show the mean proportion of the reports by cultivar by user, and the total reports by cultivar. It should be noted that for all partitions, individual users typically gave a 1% of the reports of any specific cultivar.

Supplementary Figure 1: Reports per subject per cultivar

Supplementary Figure 1 shows which fraction of the strains (N total = 887) presents a maximum fraction of reports given by only one subject. In the perfect case, we expect that all strains had only one report. In this case, the area under the line should be 0. In the worst case, all the reports by each strain were given by only one subject with an area of 1. The data from Leafly.com by August 2018 has an area under the curve of 0.05. As shown in the figure 1, only 10% of the studied cultivars presented more than 10% of their reports given by a single user, and this was reduced to 0.6% of varieties with more than 20% reports by a single user, which suggested that bias by single user reports could impact a reduced sample of the whole ensemble.

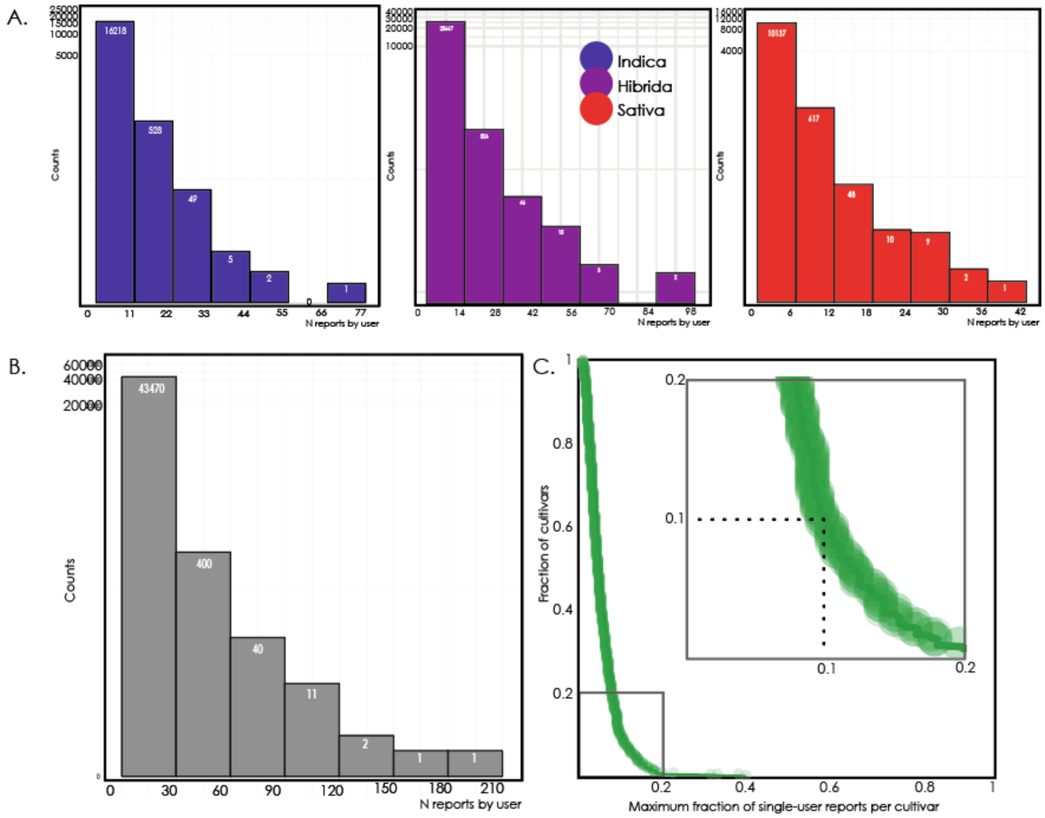

Supplementary Figure 1: Reports per subject per cultivar

A. Histograms of the count of subjects with N number of reports per for different "species" tag. B. Histograms of the count of subjects with N number of reports in all reports. It should be noted that for some subjects with a large number of reports, the reports correspond to different "species" tags. C. Validation of the reports per subject per cultivar. Fraction of cultivars vs the maximum fraction of single-user reports per cultivar.

Supplementary Figure 2: Example report by one cultivar.

Example report from Leafly.com (August 2018). It should be noted that in relation to the current information displayed in Leafly.com, no data regarding THC/CBD content, terpenes and strain origin was

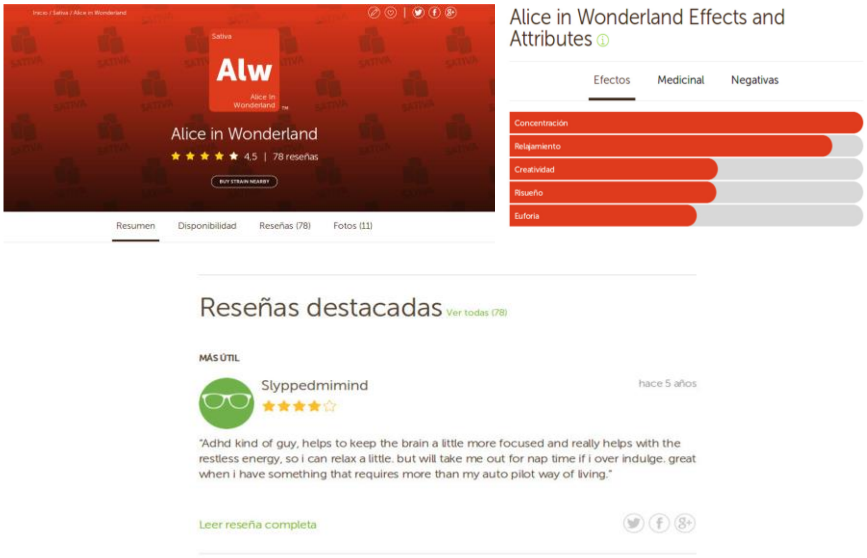

Supplementary Figure 2: Example report by one cultivar.
Format from August 2018

available at the time the data was retrieved.

.

Supplementary Table 1: Strains

Supplementary Table 1: Strains

Includes species, number of reports (N rep.) and terpene content availability (Ter.)

Strain	Species	N rep.	Ter.	Strain	Species	N rep.	Ter.
1024	Sativa	26	NO	kushage	Hybrid	14	NO
24k-gold	Hybrid	135	YES	kushberry	Indica	115	NO
3-kings	Hybrid	217	NO	la-chocolat	Hybrid	43	NO
3x-crazy	Indica	94	NO	la-confidential	Indica	853	NO
501st-og	Hybrid	79	YES	lake-of-fire	Hybrid	32	NO
5th-element	Indica	25	NO	la-kush	Hybrid	20	YES
707-headband	Hybrid	227	NO	lamb-s-bread	Sativa	571	NO
818-og	Indica	17	NO	la-og	Indica	40	YES
8-ball-kush	Indica	33	NO	larry-og	Hybrid	516	YES
a-10	Indica	76	NO	laughing-buddha	Sativa	135	NO
abusive-og	Indica	108	NO	la-ultra	Indica	12	NO
acdc	Hybrid	555	YES	lavender-haze	Sativa	13	NO
ace-of-spades	Indica	234	NO	la-woman	Hybrid	64	NO
acid	Hybrid	14	NO	lee-roy	Indica	15	NO
a-dub	Hybrid	43	NO	lemon-alien-dawg	Hybrid	46	YES
afgahni-bullrider	Indica	72	NO	lemonberry	Hybrid	28	NO
afghan-diesel	Hybrid	13	NO	lemon-diesel	Hybrid	379	NO
afghan-haze	Hybrid	37	NO	lemon-drop	Hybrid	126	NO
afghani	Indica	355	YES	lemon-g	Sativa	129	YES
afghan-skunk	Indica	28	NO	lemonhead-og	Hybrid	48	NO
afgoo	Indica	438	NO	lemon-jack	Sativa	83	YES
afgooey	Indica	244	NO	lemon-meringue	Sativa	75	NO
african	Sativa	28	NO	lemon-og	Hybrid	297	YES
afternoon-delight	Hybrid	13	YES	lemon-og-haze	Hybrid	22	NO
agent-orange	Hybrid	813	YES	lemon-pie	Sativa	24	NO
alaska	Sativa	37	NO	lemon-skunk	Hybrid	607	YES
alaskan-ice	Sativa	55	NO	lemon-thai	Sativa	86	NO
albert-walker	Hybrid	52	NO	lemon-wreck	Hybrid	34	NO
alice-in-wonderland	Sativa	65	NO	liberty-haze	Hybrid	188	NO
alien-asshat	Hybrid	14	NO	lifesaver	Hybrid	20	NO
alien-bubba	Indica	32	NO	lime-skunk	Sativa	41	YES
alien-dawg	Indica	141	NO	limon	Sativa	11	NO
alien-kush	Indica	80	YES	locktite	Hybrid	43	NO
alien-rift	Indica	21	NO	locomotion	Indica	25	YES
alien-rock-candy	Hybrid	117	NO	lodi-dodi	Hybrid	85	NO
aliens-on-moonshine	Indica	12	NO	lost-coast-og	Hybrid	57	NO
allen-wrench	Sativa	129	YES	loud-dream	Hybrid	26	NO
aloha	Sativa	23	NO	love-potion	Sativa	83	NO
alpha-blue	Sativa	70	NO	love-potion-9	Indica	26	NO
alpha-og	Hybrid	25	NO	lsd	Hybrid	748	YES
alpine-star	Indica	11	NO	lucid-dream	Hybrid	57	NO
ambrosia	Hybrid	51	NO	lucky-charms	Hybrid	107	NO
amnesia	Sativa	448	YES	lucy	Hybrid	22	YES
amnesia-haze	Sativa	888	YES	mad-scientist	Indica	20	NO
ancient-og	Indica	56	YES	magnum	Hybrid	18	NO
anesthesia	Indica	22	NO	malawi	Sativa	51	YES
apollo-11	Hybrid	31	NO	mammoth	Hybrid	15	NO
apollo-13	Hybrid	95	YES	mango	Indica	321	NO
apple-jack	Hybrid	28	NO	mango-haze	Sativa	161	NO
apple-kush	Hybrid	27	NO	mango-tango	Hybrid	54	NO

arjans-strawberry-haze	Sativa	76	NO	maple-leaf-indica	Indica	22	NO
atomic-northern-lights	Hybrid	74	NO	marionberry-kush	Hybrid	126	YES
a-train	Hybrid	50	NO	martian-mean-green	Hybrid	48	NO
aurora-borealis	Indica	19	NO	master-skunk	Hybrid	13	NO
aurora-indica	Indica	65	NO	matanuska-thunder-fuck	Sativa	136	NO
b-52	Indica	19	NO	mataro-blue	Indica	12	NO
banana-og	Hybrid	298	YES	maui	Sativa	90	NO
bananas	Hybrid	18	NO	mazar-i-sharif	Indica	88	NO
barbara-bud	Indica	20	NO	mazar-kush	Indica	41	YES
bay-11	Sativa	63	NO	medibud	Hybrid	77	NO
bay-dream	Sativa	74	NO	medicine-man	Hybrid	96	NO
bc-big-bud	Hybrid	44	NO	medicine-woman	Hybrid	11	NO
beast-mode-og	Hybrid	24	NO	medihaze	Sativa	105	NO
berry-bomb	Hybrid	39	YES	medusa	Hybrid	40	NO
berry-og	Hybrid	38	NO	melon-gum	Hybrid	28	NO
berry-white	Indica	713	NO	memory-loss	Sativa	29	NO
bettie-page	Hybrid	21	NO	mendo-breath	Indica	167	NO
big-bang	Indica	36	NO	mendocino-purps	Hybrid	203	YES
big-bud	Indica	203	NO	mickey-kush	Sativa	82	YES
big-budda-cheese	Hybrid	173	NO	middlefork	Hybrid	90	NO
big-wreck	Indica	32	NO	midnight	Hybrid	73	NO
bio-diesel	Hybrid	145	NO	mk-ultra	Indica	458	NO
black-84	Indica	19	NO	mob-boss	Hybrid	186	NO
black-afghan	Indica	22	YES	money-maker	Indica	39	YES
blackberry	Hybrid	387	YES	monkey-paw	Hybrid	26	YES
blackberry-bubble	Indica	14	NO	monster-cookies	Indica	199	YES
blackberry-cream	Indica	15	YES	montana-silvertip	Hybrid	83	NO
blackberry-kush	Indica	1341	YES	moonshine-haze	Sativa	94	YES
blackberry-rhino	Indica	15	NO	moose-and-lobsta	Sativa	42	NO
blackberry-trainwreck	Hybrid	11	YES	narnia	Sativa	41	NO
black-betty	Hybrid	60	NO	nebula	Hybrid	198	NO
black-cherry-cheesecake	Hybrid	39	NO	nepalese	Sativa	27	NO
black-cherry-og	Indica	57	NO	nevilles-haze	Sativa	59	NO
black-cherry-soda	Hybrid	250	NO	nightfire-og	Sativa	17	NO
black-diamond	Indica	211	NO	night-terror-og	Indica	73	NO
black-diesel	Sativa	42	NO	nordle	Indica	43	NO
black-domina	Indica	208	YES	northernberry	Indica	36	NO
black-haze	Hybrid	24	NO	northern-lights--5	Indica	131	NO
black-jack	Hybrid	259	NO	northern-lights-5-x-haze	Sativa	25	NO
black-lime-special-reserve	Indica	88	NO	northern-wreck	Indica	66	NO
black-russian	Indica	51	NO	nuken	Indica	150	NO
blackwater	Indica	398	YES	nyc-diesel	Hybrid	628	YES
black-widow	Hybrid	217	NO	nypd	Sativa	35	NO
blockhead	Hybrid	46	NO	obama-kush	Indica	505	NO
blue-afghani	Indica	28	NO	og-cheese	Hybrid	60	NO
blue-bastard	Indica	12	NO	og-chem	Hybrid	101	NO
blueberry	Indica	1456	YES	og-diesel-kush	Hybrid	68	NO
blueberry-ak	Hybrid	15	NO	ogiesel	Hybrid	43	YES
blueberry-blast	Sativa	13	NO	og-la-affie	Indica	16	NO
blueberry-cheesecake	Hybrid	165	YES	og-poison	Hybrid	24	NO
blueberry-diesel	Hybrid	222	NO	og-skunk	Hybrid	28	NO
blueberry-dream	Sativa	28	NO	omega	Hybrid	20	NO
blueberry-haze	Hybrid	212	YES	orange-bud	Hybrid	138	YES
blueberry-headband	Hybrid	266	YES	orange-cookies	Hybrid	169	YES
blueberry-jack	Hybrid	47	NO	orange-creamsicle	Hybrid	44	YES
blueberry-muffins	Hybrid	49	NO	orange-crush	Hybrid	345	YES
blueberry-og	Hybrid	27	NO	orange-diesel	Hybrid	53	YES
blueberry-pancakes	Hybrid	30	NO	orange-haze	Hybrid	90	NO
blueberry-pie	Hybrid	45	NO	orange-juice	Hybrid	18	NO
blueberry-silvertip	Hybrid	33	NO	orange-skunk	Hybrid	15	NO
blueberry-skunk	Indica	64	NO	orange-velvet	Hybrid	38	NO
blueberry-yum-yum	Hybrid	85	NO	oregon-diesel	Indica	65	NO
blue-boy	Hybrid	39	NO	outer-space	Sativa	56	NO
blue-champange	Hybrid	36	NO	p-51	Indica	11	NO
blue-cookies	Hybrid	281	NO	pakistani-chitral-kush	Indica	33	NO
blue-crack	Hybrid	97	NO	panama-red	Sativa	125	NO
blue-diamond	Indica	85	NO	pandoras-box	Sativa	97	NO
blue-diesel	Hybrid	404	YES	papaya	Indica	123	NO
blue-dot	Sativa	25	NO	paris-og	Indica	128	NO
blue-dragon	Hybrid	180	NO	peaches-and-cream	Hybrid	40	NO

blue-dynamite	Indica	31	NO
blue-frost	Hybrid	83	NO
blue-god	Indica	98	NO
blue-hash	Hybrid	25	NO
blue-hawaiian	Hybrid	130	NO
blue-haze	Hybrid	199	NO
blue-knight	Indica	69	NO
blue-kush	Hybrid	62	NO
blue-lights	Indica	33	NO
blue-magoo	Hybrid	267	NO
blue-moon-rocks	Hybrid	64	NO
blue-moonshine	Indica	58	NO
blue-mystic	Indica	96	NO
blue-nightmare	Hybrid	27	NO
blue-og	Hybrid	104	NO
blue-power	Indica	38	NO
blue-rhino	Hybrid	83	NO
blues	Hybrid	18	NO
blue-satellite	Sativa	35	NO
blue-train	Hybrid	44	NO
blue-venom	Hybrid	47	NO
blue-widow	Hybrid	272	NO
blurple	Hybrid	14	NO
boggle-gum	Hybrid	12	NO
boss-hogg	Hybrid	39	NO
brainstorm-haze	Sativa	29	NO
brand-x	Indica	47	NO
brian-berry-cough	Hybrid	19	NO
bruce-banner	Hybrid	737	YES
bsc	Hybrid	31	NO
bubba-og	Indica	213	NO
bubbas-gift	Indica	46	NO
bubbleberry	Hybrid	107	NO
bubble-gum	Hybrid	691	YES
bubblegum-kush	Indica	227	YES
bubblegun	Hybrid	17	NO
bubblicious	Hybrid	42	YES
buddhas-sister	Indica	96	NO
buddha-tahoe	Indica	61	NO
burkle	Indica	22	NO
burmese-kush	Hybrid	118	NO
cactus	Indica	82	NO
california-orange	Hybrid	233	NO
cali-kush	Hybrid	163	NO
cannalope-haze	Sativa	160	NO
cannalope-kush	Hybrid	97	NO
cannatonic	Hybrid	637	YES
canna-tsu	Hybrid	121	NO
caramel-candy-kush	Hybrid	44	NO
carl-sagan	Hybrid	13	NO
casey-jones	Sativa	315	NO
cataract-kush	Hybrid	135	NO
cbd-critical-cure	Indica	82	NO
cbd-kush	Hybrid	35	NO
cbd-mango-haze	Sativa	21	NO
cbd-shark	Indica	64	NO
charlie-sheen	Hybrid	111	NO
charlottes-web	Sativa	168	NO
cheese-candy	Hybrid	43	NO
cheese-quake	Hybrid	204	YES
cheesewreck	Hybrid	47	NO
chemdawg-4	Hybrid	241	YES
chemdawg-91	Hybrid	120	YES
chemdawg-sour-diesel	Hybrid	68	YES
chemmy-jones	Hybrid	49	NO
chemo	Indica	181	NO
chem-valley-kush	Hybrid	65	NO
chemwreck	Hybrid	11	NO
chernobyl	Hybrid	541	YES
cherry-ak-47	Hybrid	187	NO
pearl-scout-cookies	Hybrid	36	NO
pennywise	Indica	283	YES
peppermint-cookies	Hybrid	32	NO
permafrost	Hybrid	277	YES
pez	Indica	26	NO
phantom-cookies	Hybrid	162	YES
pie-face-og	Hybrid	45	NO
pineapple	Hybrid	334	NO
pineapple-diesel	Hybrid	55	NO
pineapple-dog-shit	Hybrid	14	NO
pineapple-fields	Sativa	21	NO
pineapple-haze	Sativa	51	NO
pineapple-jack	Sativa	75	NO
pineapple-og	Sativa	51	NO
pineapple-punch	Sativa	17	NO
pineapple-skunk	Hybrid	67	YES
pineapple-super-silver-haze	Sativa	31	NO
pineapple-thai	Sativa	57	NO
pink-champagne	Indica	37	NO
pink-cookies	Hybrid	47	NO
pink-kush	Hybrid	413	NO
pink-panther	Hybrid	57	YES
pitbull	Indica	128	NO
platinum-bubba-kush	Indica	267	NO
platinum-kush	Indica	475	YES
platinum-wreck	Hybrid	65	NO
plushberry	Indica	212	YES
pot-of-gold	Indica	115	NO
power-kush	Indica	80	NO
power-plant	Sativa	227	YES
pre-98-bubba-kush	Indica	386	YES
predator-pink	Hybrid	15	NO
presidential-og	Indica	267	NO
primus	Indica	72	NO
professor-chaos	Hybrid	21	YES
pure-afghan	Indica	35	NO
pure-kush	Indica	223	NO
pure-power-plant	Hybrid	162	NO
purple-afghani	Indica	65	NO
purple-ak-47	Hybrid	52	NO
purple-alien-og	Hybrid	86	NO
purple-arrow	Hybrid	69	NO
purple-berry	Indica	60	NO
purple-bubba	Indica	19	NO
purple-candy	Indica	186	NO
purple-champagne	Sativa	27	NO
purple-cheddar	Indica	15	NO
purple-chemdawg	Indica	99	NO
purple-crack	Hybrid	47	NO
purple-cream	Indica	50	NO
purple-diesel	Hybrid	341	YES
purple-dragon	Indica	109	NO
purple-dream	Hybrid	193	NO
purple-elephant	Indica	79	YES
purple-goo	Indica	47	NO
purple-headband	Hybrid	23	NO
purple-ice	Hybrid	19	NO
purple-jack	Hybrid	27	NO
purple-mr-nice	Indica	96	NO
purple-nepal	Indica	44	NO
purple-og-kush	Indica	241	YES
purple-panty-dropper	Indica	33	NO
purple-paralysis	Hybrid	18	NO
purple-passion	Indica	35	NO
purple-princess	Hybrid	50	NO
purple-punch	Indica	147	YES
purple-rhino	Hybrid	15	NO
purple-sour-diesel	Hybrid	178	NO
purple-star	Indica	12	NO
purple-tangie	Sativa	23	YES

cherry-bomb	Hybrid	104	NO	purple-thai	Sativa	23	NO
cherry-cookies	Hybrid	19	YES	purple-trainwreck	Hybrid	458	YES
cherry-cream-pie	Hybrid	14	NO	purple-widow	Hybrid	16	NO
cherry-diesel	Hybrid	79	NO	qleaner	Hybrid	15	NO
cherry-kola	Indica	46	NO	qrazy-train	Hybrid	127	NO
cherry-kush	Hybrid	214	NO	quantum-kush	Sativa	186	YES
cherry-og	Hybrid	99	NO	querkle	Indica	173	NO
cherry-pie	Hybrid	1225	YES	rainbow	Hybrid	79	NO
cherry-skunk	Hybrid	52	NO	rare-darkness	Indica	99	NO
chiesel	Hybrid	151	NO	raspberry-cough	Sativa	58	NO
chloe	Hybrid	19	NO	raspberry-kush	Indica	259	NO
chocolate-chunk	Indica	134	NO	recon	Indica	64	NO
chocolate-diesel	Sativa	22	NO	red-congolese	Sativa	154	NO
chocolate-fondue	Sativa	17	NO	red-diesel	Hybrid	47	NO
chocolate-hashberry	Hybrid	65	NO	red-headed-stranger	Sativa	100	NO
chocolate-kush	Indica	51	NO	red-poison	Hybrid	20	NO
chocolate-thai	Sativa	75	NO	redwood-kush	Indica	155	NO
chocolope	Sativa	904	YES	remedy	Indica	72	NO
chronic	Hybrid	112	NO	rene	Hybrid	16	NO
chronic-thunder	Indica	59	YES	ringos-gift	Hybrid	85	NO
chunky-diesel	Hybrid	25	NO	ripped-bubba	Hybrid	59	YES
church-og	Indica	78	NO	rocklock	Indica	32	NO
cinderella-99	Hybrid	686	NO	rollex-og-kush	Indica	31	NO
cinderellas-dream	Hybrid	75	NO	romulan	Indica	594	YES
citrix	Hybrid	64	NO	root-beer-kush	Sativa	19	NO
citrus-kush	Hybrid	56	NO	royal-haze	Sativa	12	NO
citrus-sap	Hybrid	87	YES	royal-kush	Hybrid	131	NO
clementine	Sativa	177	YES	rudeboi-og	Hybrid	45	NO
cloud-9	Hybrid	30	NO	rug-burn-og	Hybrid	91	YES
cold-creek-kush	Hybrid	40	NO	sage	Hybrid	180	NO
colombian-gold	Sativa	92	NO	sage-n-sour	Sativa	146	YES
confidential-cheese	Indica	100	NO	salmon-river-og	Indica	18	YES
connie-chung	Hybrid	46	NO	satori	Hybrid	44	NO
conspiracy-kush	Indica	101	YES	scooby-snack	Hybrid	120	NO
cookie-dough	Hybrid	31	NO	scotts-og	Hybrid	83	YES
cookie-monster	Indica	113	NO	seattle-cough	Sativa	34	NO
cookies-and-cream	Hybrid	280	YES	secret-recipe	Hybrid	38	NO
cookies-kush	Indica	177	YES	sensi-star	Indica	434	YES
cookie-wreck	Hybrid	72	NO	sfv-og	Hybrid	424	YES
cornbread	Indica	91	NO	sfv-og-kush	Indica	146	NO
crater-lake	Hybrid	14	NO	shangri-la	Hybrid	15	NO
crazy-miss-hyde	Hybrid	13	NO	sharks-breath	Hybrid	53	NO
cream-caramel	Indica	54	NO	shark-shock	Indica	163	NO
critical-cheese	Hybrid	33	NO	shiatsu-kush	Hybrid	37	NO
critical-haze	Hybrid	40	NO	shishkaberry	Indica	218	NO
critical-hog	Indica	51	NO	shiva-skunk	Indica	67	NO
critical-jack	Hybrid	124	NO	silver-bubble	Hybrid	34	NO
critical-kush	Indica	448	YES	silver-surfer	Hybrid	42	NO
critical-mass	Indica	536	YES	silver-train	Sativa	13	NO
critical-plus	Hybrid	125	NO	sinmint-cookies	Hybrid	57	NO
critical-sensi-star	Indica	71	NO	sin-valley-og	Hybrid	27	NO
crunch-berry-kush	Hybrid	18	NO	sirius-black	Indica	35	NO
cuvee	Indica	29	YES	skunk-1	Hybrid	309	NO
dairy-queen	Hybrid	131	YES	skunk-47	Indica	17	NO
dancehall	Hybrid	28	NO	skunkberry	Hybrid	47	NO
dance-world	Sativa	17	NO	skunk-haze	Hybrid	52	NO
dark-side-of-the-moon	Indica	36	NO	skunky-diesel	Hybrid	11	NO
darth-vader-og	Indica	105	YES	sleestack	Sativa	13	NO
deadhead-og	Hybrid	367	YES	snoop-s-dream	Hybrid	252	NO
deep-purple	Indica	55	NO	snow-bud	Hybrid	25	NO
deep-sleep	Indica	50	NO	snowcap	Hybrid	356	NO
delahaze	Sativa	52	NO	snowland	Hybrid	19	NO
diablo	Indica	196	NO	snow-leopard	Indica	35	NO
diamond-og	Indica	249	NO	snow-monster	Indica	15	NO
dirty-girl	Sativa	123	NO	snow-white	Hybrid	103	NO
dj-short-blueberry	Indica	193	YES	somango	Indica	92	YES
dog-shit	Hybrid	23	NO	sonoma-coma	Sativa	13	NO
dogwalker-og	Hybrid	181	NO	soul-assassin-og	Hybrid	22	NO
do-si-dos	Indica	230	YES	sour-alien	Hybrid	74	NO
double-dream	Hybrid	258	NO	sour-amnesia	Sativa	71	NO

double-og	Indica	12	NO	sour-banana-sherbet	Hybrid	99	YES
double-tangie-banana	Hybrid	85	YES	sour-bubba	Indica	14	NO
dragon-og	Hybrid	12	NO	sour-bubble	Indica	26	NO
dragons-breath	Hybrid	56	NO	sour-candy	Hybrid	22	NO
dream-beaver	Sativa	31	NO	sour-chees	Hybrid	78	NO
dream-berry	Indica	22	NO	sour-cookies	Hybrid	53	NO
dream-lotus	Hybrid	25	NO	sour-cream	Hybrid	63	NO
dr-funk	Indica	24	NO	sour-dream	Hybrid	90	NO
dr-grinspoon	Sativa	39	YES	sour-grape	Hybrid	131	YES
dr-who	Hybrid	352	NO	sour-grapes	Hybrid	270	NO
durban-berry	Hybrid	11	NO	sour-haze	Sativa	46	NO
durban-cookies	Sativa	39	NO	sour-headband	Hybrid	16	NO
dutchberry	Hybrid	71	NO	sour-jack	Sativa	122	NO
dutch-dragon	Sativa	66	NO	sour-kush	Hybrid	429	YES
dutch-hawaiian	Sativa	100	NO	sour-lemon-og	Hybrid	62	NO
dutch-queen	Hybrid	12	NO	sourlope	Sativa	19	YES
dutch-treat	Hybrid	749	NO	sour-lsd	Hybrid	16	NO
dutch-treat-haze	Hybrid	38	YES	sour-maui	Sativa	31	NO
dynamite	Indica	63	NO	sour-og	Hybrid	482	YES
early-girl	Indica	40	NO	sour-patch-kiss	Hybrid	11	NO
earth-og	Hybrid	32	NO	sour-pebbles	Sativa	23	NO
east-coast-sour-diesel	Sativa	135	YES	sour-power	Hybrid	23	YES
elderberry-kush	Hybrid	17	NO	sour-tangie	Sativa	330	YES
electric-lemon-g	Sativa	33	NO	sour-tsunami	Hybrid	172	NO
el-jeffe	Indica	53	NO	sour-urkle	Hybrid	39	NO
el-nino	Hybrid	12	NO	space-bomb	Hybrid	55	NO
elvis	Hybrid	28	NO	space-candy	Hybrid	68	YES
emerald-jack	Sativa	57	NO	space-dawg	Indica	27	NO
emerald-og	Indica	36	NO	starbud	Indica	43	NO
enigma	Indica	14	NO	stardawg	Hybrid	334	YES
eran-almog	Indica	19	NO	stardawg-guava	Sativa	25	NO
euphoria	Sativa	83	NO	starfighter	Hybrid	30	NO
exodus-cheese	Hybrid	95	YES	star-killer	Indica	159	YES
extreme-cream	Indica	21	YES	stephen-hawking-kush	Indica	29	NO
face-off-og	Indica	66	NO	strawberry	Sativa	134	NO
fire-alien-kush	Hybrid	30	NO	strawberry-amnesia	Sativa	27	NO
flo	Hybrid	417	NO	strawberry-blue	Sativa	38	YES
flo-og	Indica	28	YES	strawberry-cheesecake	Indica	100	NO
flowerbomb-kush	Indica	33	NO	strawberry-diesel	Hybrid	192	YES
fortune-cookies	Hybrid	99	NO	strawberry-dream	Hybrid	35	NO
frankenstein	Indica	53	NO	strawberry-fields	Indica	94	NO
franks-gift	Hybrid	33	NO	strawberry-ice	Sativa	27	NO
freezeland	Indica	21	NO	strawberry-kush	Hybrid	248	NO
frida	Indica	12	NO	strawberry-lemonade	Sativa	133	NO
frisian-dew	Hybrid	18	NO	strawberry-mango-haze	Sativa	16	NO
frostbite	Sativa	16	NO	strawberry-og	Hybrid	17	YES
frosted-freak	Hybrid	60	NO	sugar-black-rose	Indica	149	YES
fruit-loops	Hybrid	43	NO	sugar-cookie	Hybrid	155	YES
fruit-punch	Sativa	61	YES	sugar-kush	Indica	41	NO
fruit-spirit	Hybrid	23	NO	sugar-plum	Sativa	38	NO
fruity-chronic-juice	Indica	21	YES	sugar-punch	Hybrid	11	NO
fucking-incredible	Indica	178	NO	sunshine	Sativa	38	YES
funfetti	Hybrid	19	NO	sunshine-daydream	Indica	51	YES
future	Hybrid	38	NO	super-blue-dream	Hybrid	150	NO
g13-haze	Hybrid	132	NO	super-green-crack	Sativa	64	YES
galactic-jack	Sativa	72	YES	super-jack	Sativa	132	NO
game-changer	Hybrid	76	NO	super-kush	Indica	55	NO
gelato	Hybrid	678	YES	super-lemon-haze	Sativa	1373	YES
ghost-og	Hybrid	381	NO	super-lemon-og	Hybrid	47	NO
ghost-train-haze	Sativa	640	YES	superman-og	Indica	144	NO
gigabud	Indica	28	YES	supermax-og	Hybrid	32	NO
glass-slipper	Hybrid	166	NO	supernova	Hybrid	27	NO
godberry	Indica	63	NO	super-silver-haze	Sativa	312	YES
god-bud	Indica	439	NO	super-skunk	Indica	365	YES
godfather-og	Indica	117	NO	super-snow-dog	Sativa	73	NO
godzilla	Indica	22	NO	super-sour-diesel	Sativa	361	YES
goji-og	Hybrid	153	YES	super-sour-og	Hybrid	28	YES
golden-goat	Hybrid	871	YES	super-sour-skunk	Hybrid	32	NO
golden-lemon	Hybrid	32	NO	super-sour-widow	Hybrid	12	NO
golden-pineapple	Hybrid	314	NO	super-sweet	Hybrid	41	NO

golden-ticket	Hybrid	97	NO	suzy-q	Hybrid	21	NO
goo	Indica	129	NO	sweet-and-sour-widow	Indica	29	NO
gooberry	Indica	22	NO	sweet-baby-jane	Indica	15	NO
gorilla-cookies	Hybrid	70	YES	sweet-black-angel	Indica	21	NO
grandpa-larry-og	Indica	33	NO	sweet-cheese	Sativa	63	NO
grape-cookies	Indica	16	NO	sweet-deep-grapefruit	Indica	14	NO
grapefruit	Sativa	372	NO	sweet-diesel	Sativa	48	NO
grapefruit-diesel	Hybrid	92	NO	sweet-dreams	Hybrid	25	NO
grapefruit-haze	Sativa	35	NO	sweet-kush	Hybrid	62	NO
grapefruit-kush	Hybrid	127	NO	sweet-tooth	Indica	411	NO
grape-god	Hybrid	307	YES	swiss-tsunami	Sativa	11	NO
grape-inferno	Indica	13	NO	tahoe-alien	Hybrid	39	NO
grape-krush	Indica	98	NO	tahoe-og	Hybrid	232	YES
grape-kush	Hybrid	96	NO	tangerine	Hybrid	137	NO
grape-ox	Indica	24	NO	tangerine-dream	Hybrid	615	YES
grape-skunk	Indica	23	NO	tangerine-haze	Hybrid	147	YES
grape-valley-kush	Indica	14	NO	tangerine-kush	Indica	168	NO
grease-monkey	Hybrid	108	YES	tangerine-power	Hybrid	68	YES
green-dragon	Indica	52	NO	tangilope	Sativa	91	YES
green-dream	Hybrid	120	NO	thai	Sativa	94	NO
green-goddess	Hybrid	49	NO	thai-girl	Hybrid	14	NO
green-kush	Indica	27	NO	thai-haze	Sativa	12	NO
green-lantern	Sativa	19	NO	thai-lights	Hybrid	22	NO
green-love-potion	Indica	17	NO	the-black	Indica	54	NO
green-poison	Indica	89	NO	the-cough	Sativa	15	NO
green-queen	Hybrid	133	NO	the-doctor	Indica	17	NO
green-ribbon	Hybrid	106	NO	the-grunk	Hybrid	31	NO
griz-kush	Hybrid	25	NO	the-one	Hybrid	16	NO
guava-chem	Hybrid	25	NO	the-sauce	Hybrid	39	NO
guava-kush	Hybrid	58	NO	the-third-dimension	Hybrid	14	NO
gumbo	Indica	36	NO	the-truth	Hybrid	62	NO
gummy-bears	Hybrid	21	NO	the-void	Hybrid	43	NO
haole	Hybrid	25	NO	the-white	Hybrid	258	YES
harlequin	Sativa	877	NO	think-different	Hybrid	15	NO
harle-tsu	Hybrid	238	YES	thin-mint	Hybrid	525	YES
hashberry	Indica	71	NO	timewreck	Sativa	98	NO
hash-plant	Indica	309	NO	tina-danza	Hybrid	20	YES
hawaiian	Sativa	103	NO	tora-bora	Indica	76	YES
hawaiian-diesel	Sativa	15	NO	training-day	Indica	41	YES
hawaiian-dream	Sativa	52	NO	tres-dawg	Hybrid	19	NO
hawaiian-fire	Hybrid	13	NO	triangle-kush	Indica	69	YES
hawaiian-haze	Sativa	64	NO	trident	Hybrid	11	NO
hawaiian-punch	Sativa	45	NO	trinity	Sativa	125	NO
hawaiian-purple-kush	Indica	24	NO	triple-cheese	Indica	12	NO
hawaiian-skunk	Hybrid	26	NO	tropicali	Hybrid	16	NO
hawaiian-snow	Sativa	130	YES	true-og	Indica	308	NO
haze	Sativa	281	YES	tuna-kush	Indica	43	NO
headbanger	Hybrid	49	YES	tutankhamon	Sativa	138	YES
head-cheese	Hybrid	140	NO	tutti-frutti	Hybrid	50	NO
head-trip	Hybrid	18	NO	twisted-citrus	Sativa	11	NO
heavy-duty-fruity	Hybrid	56	YES	u2-kush	Indica	22	NO
heisenberg-kush	Sativa	21	NO	uk-cheese	Hybrid	416	YES
hellfire-og	Hybrid	53	NO	ultraviolet-og	Indica	19	NO
hell-raiser-og	Hybrid	22	NO	uw	Indica	85	NO
hempstar	Sativa	84	NO	vader-og	Indica	13	NO
hindu-skunk	Indica	76	NO	valentine-x	Hybrid	11	NO
hippie-crippler	Hybrid	84	NO	valley-girl	Hybrid	22	NO
hog	Indica	82	NO	vanilla-kush	Indica	348	YES
hollands-hope	Indica	40	NO	vcdc	Hybrid	29	NO
hollywood-og	Hybrid	37	NO	venice-og	Sativa	19	NO
honey-bananas	Hybrid	94	NO	venom-og	Hybrid	104	YES
honey-boo-boo	Indica	20	NO	very-berry-haze	Sativa	35	NO
huckleberry	Hybrid	109	NO	violator-kush	Indica	216	NO
humboldt	Hybrid	25	NO	voodoo	Sativa	40	NO
hurkle	Hybrid	39	NO	vortex	Sativa	172	YES
hurricane	Sativa	29	YES	walker-kush	Hybrid	20	NO
ice	Hybrid	157	NO	walter-white	Hybrid	39	NO
ice-cream	Hybrid	104	YES	wappa	Hybrid	84	NO
iced-grapefruit	Hybrid	42	YES	warlock	Hybrid	47	NO
iced-widow	Indica	25	NO	watermelon	Indica	123	NO

ice-wreck	Hybrid	24	NO
incredible-bulk	Indica	61	YES
incredible-hulk	Sativa	53	NO
in-the-pines	Sativa	33	YES
island-sweet-skunk	Sativa	560	NO
j1	Hybrid	466	NO
jack-diesel	Sativa	37	NO
jack-flash	Hybrid	227	NO
jack-frost	Hybrid	335	NO
jack-haze	Hybrid	27	NO
jack-kush	Hybrid	16	NO
jacks-cleaner	Sativa	48	NO
jack-skellington	Sativa	73	NO
jack-the-ripper	Sativa	302	NO
jack-wreck	Sativa	14	NO
jacky-white	Sativa	33	NO
jamaican	Sativa	30	YES
jamaican-dream	Sativa	58	NO
jamaican-lion	Sativa	11	NO
jazz	Hybrid	16	NO
jedi-kush	Indica	238	YES
jenny-kush	Hybrid	57	NO
jesus	Hybrid	37	NO
jet-fuel	Hybrid	265	NO
jr	Indica	188	NO
juicy-fruit	Hybrid	439	NO
julius-caesar	Hybrid	16	YES
k2	Hybrid	24	NO
kaboom	Sativa	47	NO
kalashnikova	Hybrid	14	NO
kandy-kush	Hybrid	368	YES
kens-kush	Hybrid	20	NO
key-lime-haze	Sativa	21	NO
key-lime-pie	Hybrid	190	NO
khalifa-kush	Hybrid	370	NO
kilimanjaro	Sativa	97	NO
killing-fields	Sativa	18	NO
kimbo-kush	Hybrid	104	NO
king-kong	Hybrid	92	NO
kong	Hybrid	103	NO
kosher-kush	Indica	601	YES
kosher-tangie	Hybrid	115	NO
kryptonite	Indica	218	NO
k-train	Indica	13	NO
wedding-cake	Hybrid	195	YES
white-99	Hybrid	42	NO
white-berry	Indica	115	NO
white-buffalo	Sativa	89	NO
white-cheese	Hybrid	29	NO
white-cookies	Hybrid	51	NO
white-dawg	Hybrid	34	NO
white-diesel	Hybrid	28	YES
white-dream	Hybrid	18	NO
white-durban	Sativa	14	NO
white-elephant	Hybrid	20	NO
white-fire-43	Indica	33	NO
white-fire-alien-og	Hybrid	78	NO
white-fire-og	Hybrid	629	YES
white-gold	Hybrid	71	NO
white-gorilla	Hybrid	21	NO
white-haze	Sativa	12	NO
white-kush	Indica	67	NO
white-lavender	Hybrid	41	NO
white-lightning	Hybrid	66	YES
white-lotus	Hybrid	16	NO
white-nightmare	Sativa	85	NO
white-og	Indica	53	YES
white-queen	Hybrid	23	NO
white-romulan	Hybrid	42	NO
white-shark	Sativa	318	NO
white-skunk	Hybrid	21	NO
white-urkle	Hybrid	55	NO
white-walker-kush	Hybrid	37	NO
whitewalker-og	Indica	63	NO
willie-nelson	Sativa	96	NO
willys-wonder	Indica	168	NO
willy-wonka	Sativa	28	NO
wonder-woman	Hybrid	110	NO
woody-kush	Indica	41	NO
wookies	Hybrid	30	NO
xxx-og	Indica	191	NO
yeti-og	Indica	39	NO
y-griega	Sativa	41	NO
yoda-og	Indica	228	NO
yumboldt	Indica	52	NO
yummy	Hybrid	17	NO
zeus-og	Hybrid	24	NO

Supplementary Table 2: Effects

Supplementary Table 2: Effects

	Hybrid (N = 451)		Indica (N = 265)		Sativa (N = 171)	
	mean	sd	mean	sd	mean	sd
Aroused	0.123	0.081	0.106	0.074	0.148	0.092
Creative	0.333	0.132	0.21	0.101	0.427	0.108
Energetic	0.296	0.148	0.116	0.076	0.503	0.141
Euphoric	0.528	0.13	0.481	0.119	0.524	0.111
Focused	0.299	0.121	0.189	0.089	0.393	0.126
Giggly	0.229	0.1	0.179	0.088	0.222	0.102
Happy	0.626	0.111	0.533	0.113	0.642	0.102
Hungry	0.251	0.109	0.288	0.114	0.183	0.091
Relaxed	0.629	0.152	0.755	0.13	0.44	0.14
Sleepy	0.204	0.12	0.449	0.146	0.088	0.072
Talkative	0.227	0.107	0.13	0.082	0.285	0.113
Tingly	0.193	0.086	0.197	0.085	0.166	0.073
Uplifted	0.491	0.136	0.336	0.115	0.586	0.121
Anxious	0.048	0.05	0.035	0.04	0.056	0.05
Dizzy	0.066	0.057	0.063	0.052	0.061	0.056
Dry.Eyes	0.152	0.084	0.162	0.077	0.137	0.079
Dry.Mouth	0.265	0.107	0.293	0.11	0.247	0.106
Headache	0.036	0.042	0.034	0.038	0.036	0.042

Paranoid	0.051	0.053	0.043	0.045	0.055	0.053

Supplementary Table 3: Flavours

sd: standard deviation

Supplementary Table 3: Flavours

sd: standard deviation

	Hybrid (N = 451)		Indica (N = 265)		Sativa (N = 171)	
	mean	sd	mean	sd	mean	sd
Ammonia	0.012	0.03	0.012	0.026	0.013	0.037
Apple	0.014	0.072	0.01	0.026	0.006	0.016
Apricot	0.008	0.032	0.007	0.03	0.005	0.014
Berry	0.139	0.182	0.201	0.228	0.076	0.125
Blue Cheese	0.008	0.024	0.008	0.039	0.006	0.026
Blueberry	0.077	0.187	0.073	0.152	0.028	0.111
Butter	0.017	0.058	0.015	0.047	0.013	0.034
Cheese	0.048	0.159	0.036	0.107	0.027	0.096
Chemical	0.04	0.07	0.027	0.051	0.02	0.037
Chestnut	0.008	0.022	0.011	0.031	0.008	0.021
Citrus	0.198	0.184	0.135	0.142	0.256	0.202
Coffee	0.017	0.054	0.02	0.047	0.023	0.071
Diesel	0.125	0.221	0.067	0.113	0.1	0.192
Earthy	0.32	0.2	0.402	0.209	0.311	0.229
Flowery	0.114	0.105	0.13	0.117	0.106	0.103
Grape	0.069	0.146	0.12	0.191	0.066	0.129
Grapefruit	0.031	0.088	0.025	0.069	0.046	0.113
Honey	0.032	0.079	0.026	0.07	0.017	0.03
Lavender	0.025	0.066	0.033	0.061	0.023	0.081
Lemon	0.108	0.174	0.065	0.108	0.136	0.212
Lime	0.034	0.07	0.025	0.071	0.046	0.11
Mango	0.021	0.067	0.017	0.084	0.037	0.128
Menthol	0.012	0.035	0.012	0.03	0.009	0.028
Mint	0.04	0.09	0.034	0.061	0.035	0.058

	Hybrid (N = 451)		Indica (N = 265)		Sativa (N = 171)	
	mean	sd	mean	sd	mean	sd
Nutty	0.03	0.049	0.044	0.075	0.031	0.058
Orange	0.052	0.154	0.019	0.045	0.046	0.094
Peach	0.011	0.05	0.005	0.018	0.008	0.022
Pear	0.007	0.033	0.009	0.025	0.006	0.018
Pepper	0.029	0.057	0.038	0.072	0.04	0.078
Pine	0.178	0.164	0.17	0.153	0.194	0.215
Pineapple	0.028	0.104	0.01	0.027	0.069	0.184
Plum	0.007	0.023	0.013	0.029	0.011	0.062
Pungent	0.19	0.146	0.222	0.152	0.158	0.126
Rose	0.012	0.034	0.015	0.053	0.013	0.044
Sage	0.026	0.055	0.028	0.044	0.025	0.075
Skunk	0.113	0.154	0.124	0.156	0.091	0.136
Spicy.Herbal	0.086	0.089	0.105	0.098	0.114	0.131
Strawberry	0.022	0.094	0.018	0.086	0.049	0.185
Sweet	0.325	0.191	0.337	0.192	0.297	0.184
Tar	0.009	0.025	0.011	0.041	0.011	0.034
Tea	0.024	0.041	0.028	0.047	0.025	0.047
Tobacco	0.01	0.024	0.013	0.028	0.011	0.03
Tree	0.037	0.06	0.038	0.079	0.042	0.067
Fruit	0.037	0.078	0.035	0.067	0.039	0.072
Tropical	0.077	0.122	0.045	0.075	0.112	0.143
Vanilla	0.025	0.067	0.023	0.074	0.014	0.058
Violet	0.01	0.027	0.017	0.054	0.007	0.02
Woody	0.12	0.122	0.165	0.138	0.122	0.129

2. Methodology

Networks and modularity

A network is a representation of the interactions (edges) between certain objects (nodes). In this work, we represented the cultivars as networks (nodes as cultivars, e.g.: s_1 and s_2) linked with connections weighted by the value of the non-parametric Spearman rank correlation coefficient between the effect / flavour vectors of nodes $E(s_1)$ and $E(s_2)$ / $F(s_1)$ and $F(s_2)$, respectively.

A common strategy to study the structure of networks is to analyze groups of nodes more densely connected between them than with other nodes in the network, commonly called the community or modular structure of the network. Given a network with i partitions, the modularity (Q) is defined as the fraction of the internal connections (e_{ii}) minus the expected value of the same quantity in a network with the same community division, but with random connections between nodes (Dorogovtsev and Mendes, 2004):

Eq. 1

$$\mathrm{Q[\{i\}]} = \sum_{\mathrm{i}} (\mathrm{e_{ii}} - \mathrm{n_i^2}) \text{ , with } \mathrm{n_i} = \sum_{\mathrm{i}} \mathrm{e_{ij}}$$

Where e_{ij} represents edges between modules *i* and *j* (including *i = j).* The modularity for a given partition reaches values between 0 and 1. The final modularity Q of a network is computed using the partition that maximizes Eq. 1. As described by Clauset and Newman (Clauset et al., 2004), this can be

Eq. 2

$$Q[\{i\}] = \frac{1}{2m} \sum_{i,j} [A_{ij} - \frac{k_i k_j}{2m}] \, \delta(c_i, c_j)$$

expressed in terms of the adjacency matrix, $A_{i,j}$ as:

Where $A_{i,j}$ represents the weight of the edges between nodes *i* and *j*, $k_i = \sum_i A_{ij}$, is the sum of the weights of the edges to the node *i*, and c_i is the community in which the node *i* is included. The δ function is 1 when *i* = *j* and zero otherwise, and m = $\frac{1}{2}\sum_{i,j} A_{ij}$.

Computing the modularity for all possible partitions of the network is usually very costly. Because of this, various algorithms (also known as "heuristics") have been developed to estimate the best partition of a network. The most frequently followed strategies can be classified either as agglomerative or corrosive. Agglomerative algorithms take isolated nodes as starting points (each representing an independent community) and, following a procedure of step-by-step merging, calculate Q until optimization of the original value is achieved. Conversely, corrosive strategies start with the whole network identified as a single community and apply a process of step-by-step pruning to optimize Q.

In this work we used the Louvain agglomerative algorithm as implemented in Gephi (Blondel et al., 2008; Lambiotte et al., 2008). The algorithm proceeds in two iterative steps. First, the algorithm starts from all the isolated nodes considered each as a single community. Then, for each node it takes into consideration the nearest nodes and their strongest connections to the node. Next, it replaces each community by the merge of these nodes and calculates the increase in the value of Q. If no gain is possible the node remains in its original community. This step continues until no movements of the nodes between communities result in improvements to the modularity. The order of the nodes that are considered by the algorithm does not have an impact in the final communities that are discovered (Blondel et al., 2008), hence we started from a random selection. The second step consists in building a new network with the community structure determined from the previous step. For this purpose, the weights of the new merged nodes are given by the sum of the weights of the links between the new communities. Links inside the community are represented by self-links with weights equal to the sum of all the internal links (Supplementary Figure 3, A). The Gephi implementation of the algorithm is based on a generalization of this process (Lambiotte et al., 2008) that allows the inclusion of a resolution parameter (γ) to determine the hierarchical structure of the network. The algorithm was applied to maximize the above-mentioned Newman's modularity with a resolution parameter γ = 1.

Random Forest

In this work we used the random forest algorithm to classify species tags given the reported frequencies of effects and flavours. Here we give a conceptual explanation of this method - for a more extensive explanation, we recommend (Hastie, 2009; James et al., 2013).

The random forest algorithm is based on a simple and intuitive method (decision tree) combined with the addition of bootstrap aggregation of multiple models. Tree-based methods segment the prediction space based on consecutive decisions over many predictors or features. Supplementary Figure 3 (panel B) shows an example where predictor variables *x* and *y* can be separated by different cuts ("splits") in the plane to predict the class of a new and unseen sample (e.g., the white dot in Supplementary Figure 3, panel B). This procedure can be represented graphically as a tree (Supplementary Figure 3, B, right). It should be noted that when a large number of features are taken into consideration, the order in which the variables are split may affect the result, since starting from a noisy variable will negatively impact on the quality of all ensuing splits. To overcome this limitation, the random forest algorithm runs several trees selecting a subset of random variables or features as input, and then aggregates the decision of all individual models. When a new sample is evaluated by the classifier, the class is determined by the vote of each individual tree.

In order to avoid overfitting, we divided our dataset into 5 equal parts and used 4 parts to train the model and the remaining part for testing (5-fold stratified cross-validation).

Latent Semantic Analysis

Latent Semantic Analysis is a natural language processing tool allowing to determine the similarity between documents from the co-occurrence patterns of the words that compose them (Landauer et al., 1998). The general idea underlying the LSA algorithm is to represent individual terms as vectors in a semantic space with reduced rank.

As described in the main manuscript text, the first step consists in the construction of an adequate terms(w)-documents(j) matrix (A_{wj}). The second step is to decompose this matrix by means of the Singular Value Decomposition (Huang and Narendra, 2008) as $A = U \times S \times \mathrm{W}$, where U contains the matrix eigenvectors, S is a diagonal matrix containing the ordered eigenvalues of AA^{T}, and W contains the eigenvectors of $A^{\mathrm{T}}A$ (see Supplementary Figure 3, C). To reduce the impact of low and non-informative correlations in the data, we reconstructed the original matrix using the 50 higher values of S ($A_{50} = U_{50} \times S_{50} \times \mathrm{W}_{50}$).

Finally, to find the topics in the data we conducted a principal component analysis (PCA) of A_{50}. PCA is based on Singular Value Decomposition and its objective is to reorganize the data identifying the orthogonal directions where its projection leads to the maximum variance (Bertoldo et al., 2004; De Lathauwer et al., 2000; Friston et al., 1996; Gao et al., 2009; Naganawa et al., 2005; Supekar et al., 2008; Tipping and Bishop, 1999). In our case, we retained the first five principal directions, leading to the identification of the first most relevant topics in the data.

Word2vec

To validate the LSA results we used a Word2Vec (Rong, 2014) model pre-trained with the Google News corpus (https://news.google.com/), comprising a vocabulary size of 3^6 unique words, and a total of 10^{10} words for training using the skip-gram neural network architecture with dimension 300 (a common choice in related literature). After training, this model can represent any word in the corpus as a vector of 300 dimensions by assigning to each unique word in the vocabulary its corresponding vector in the embedding. Vectors corresponding to words that represent similar concepts (i.e. similar semantic content) in the corpus are located close to one another in the embedding, thus it is possible to

determine the semantic distance between words by measuring the cosine of the angle between their respective vectors in the embedding (Supplementary Figure 3).

Supplementary Figure 3: Outline of the methodology

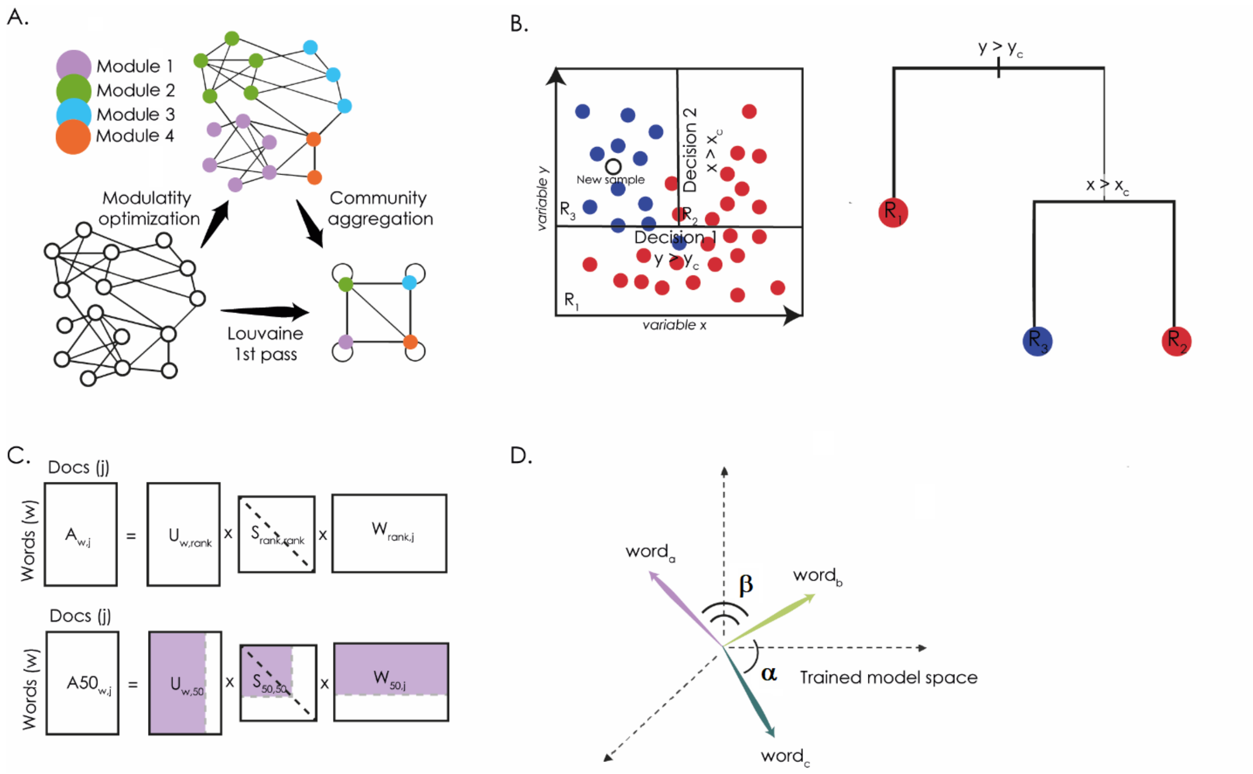

Supplementary Figure 3: outline of the methodology

A. Steps followed by the Louvain algorithm, adapted from (Blondel et al., 2008). B. Example of a partition of feature space by a decision tree. Left: splits in feature space for two features. Right: the decision tree associated with the splits presented in the left panel. C. Schematic representation of LSA and the factorization by Singular Value Decomposition. D. Three example words in a space defined by a word2vec model. The angles between the words are α and β. The semantic distance between words can be computed as the cosine distance between the associated vectors.

3. Flavors and Effects

Supplementary Figure 4: THC and CBD relation with effects

Given the observed negative association between flavours and negative effects, we explored the complementary possibility that this could be driven by THC or CBD content. To explore this possibility, we considered the 183 strains for which the cannabinoid profile is available, and computed the Spearman correlation coefficient between delta-9-THC and CBD content and the reported effects. Supplementary figure 4 shows the obtained Spearman correlations. We did not find significant

associations for THC; we found a significant positive association for CBD content and "Relaxed" effect, and negative with "Talkative", and "Paranoid" ($p < 0.01$, uncorrected, Supplementary Figure 4).

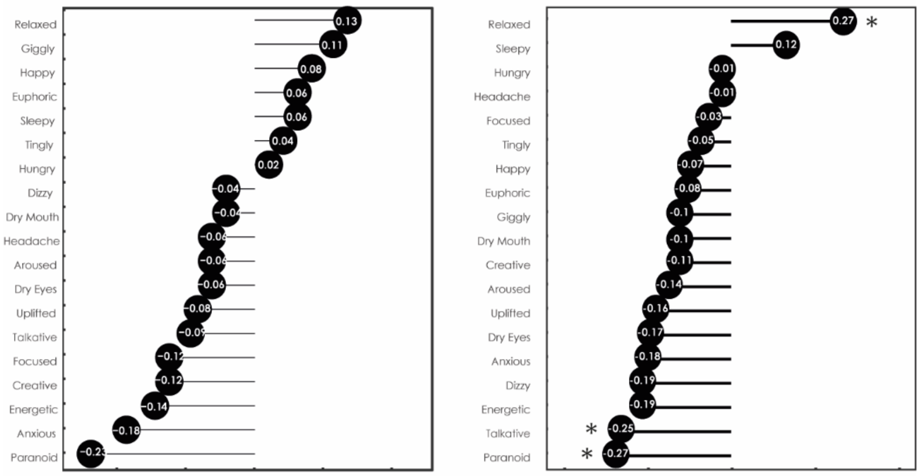

Supplementary Figure 4: THC and CBD relation with effects

Association between delta-9-THC (dry %) and CBD (dry %) with the reported effects. No significant Spearman correlations between effects and delta-9-THC (dry %) were observed ($p<0.01$, uncorrected). CBD content shows a positive relationship with "Relaxed" effect, and negative with "Talkative", and "Paranoid" ($p<0.01$, uncorrected). The figure shows the ordered correlation values.

Supplementary Figure 5: Flavors and Effects graph

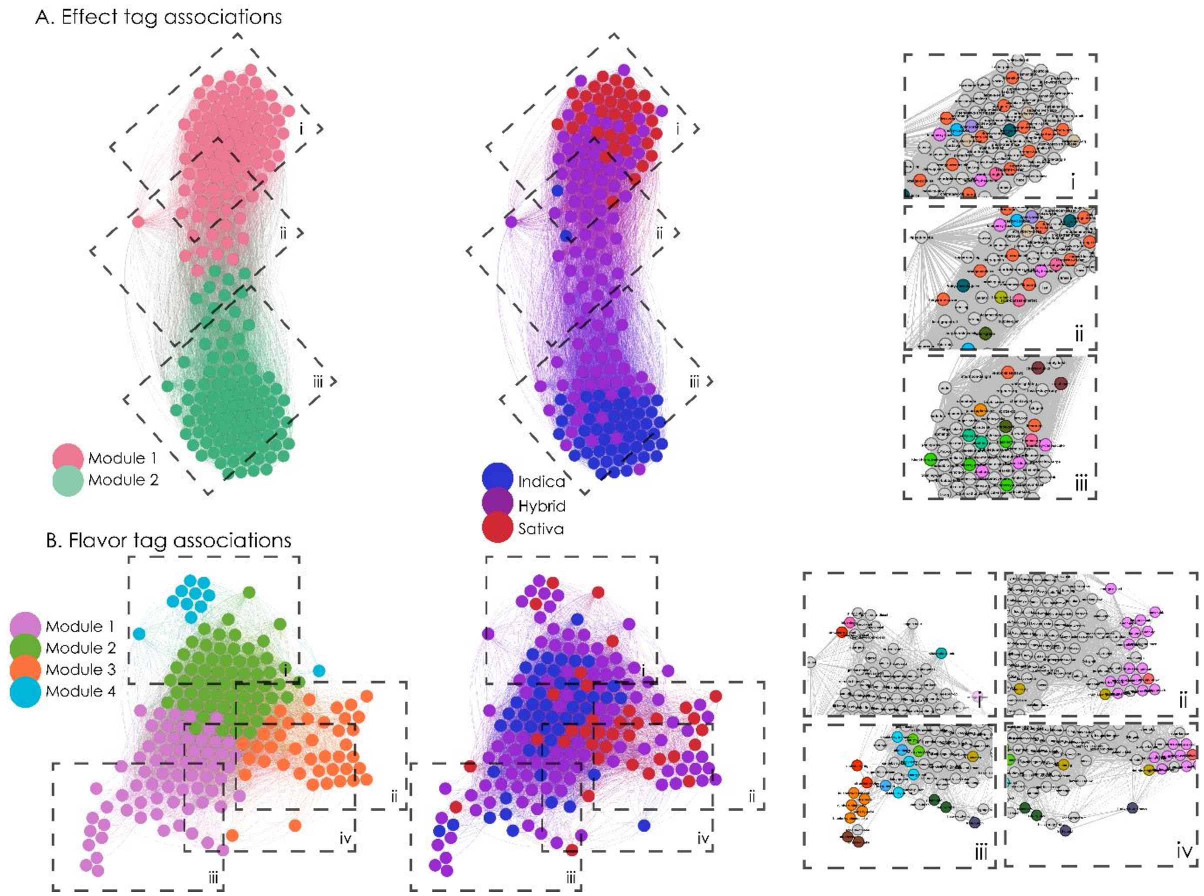

Supplementary Figure 5: Flavors and Effects graph

The restricted effects network had a modularity of 0.403, with 2 modules representing 50.27% and 49.73% of the strains. The restricted flavors network had a modularity of 0.141, with a total of 4 modules representing 37.16%, 34.43%, 22.4%, and 6.01%, respectively.

Random Forest feature importance

Supplementary table 4 presents the fraction of independent trees (1000) in which listed words appear in the top-3 more important variables for tree construction, both for the original and permuted data.

Supplementary Table 4 Feature importance

Fraction of independent trees (1000) in which a variable appears in the top-3.

Subjective effects			
Original		Permuted	
word	%	word	%
Relaxed	100	Euphoric	25.8-29.1
Hungry	100	Happy	25.9-29.2
Paranoid	100	Tingly	27.7-32.7
**Subjective flavours**			
Original		Permuted	
word	%	word	%
Earthy	100	Pungent	40.3-51
Grapefruit	100	Earthy	60.1-69
Woody	100	Sweet	62-68

4. Topics and LSA analysis

Supplementary Figure 6: LSA validation

The rank of LSA truncation was validated by the stability of the number of communities and modularity values detected using the Louvain algorithm (see Supplementary Figure 3). Given that this algorithm has random agglomerative initiation, we ran the optimization 1000 times for each low-rank frequency matrix, ranging from 10 to 60 retained eigenvalues. For each iteration, we calculated the number of communities detected (N com) and the Newman modularity of the partition (Q). Supplementary Figure 6 shows the mean number of communities and modularity across the range of retained eigenvalues. Dotted lines represent the selected rank for all the presented analyses.

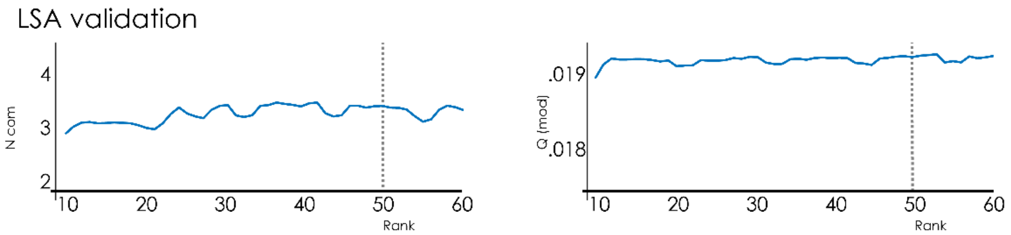

Supplementary Figure 6: LSA validation
Validation of the modularity analysis (mean across 1000 runs of the Louvain algorithm) at each rank for: Left, number of communities detected (N com) and right: modularity (Q) vs. the number of retained eigenvalues. Results are plotted with smoothing of 3 samples for visualization purposes. Dotted lines represent the selected rank for the presented analyses.

Supplementary Figure 7 Topics

PCA was applied to obtain the main topics present in the written reports. For all the reports, the variance explained by the first 5 components was 26% (Supplementary Figure 7). Upon visual inspection, we found two principal categories of topics: subjective/therapeutic effects, and plant growth/acquisition. The first component obtained from “sativa” reports consisted of a general mixture of effects, while the second was specific to therapeutic use. For the “sativa” reports, the variance explained by the first 5 components was 19%. For “indicas”, both components explain the highest variance related to therapeutic effects. For the “indica” reports, the variance explained by the first 5 components was 21%. In both cases, the rest of the components were associated with plant growth/acquisition.

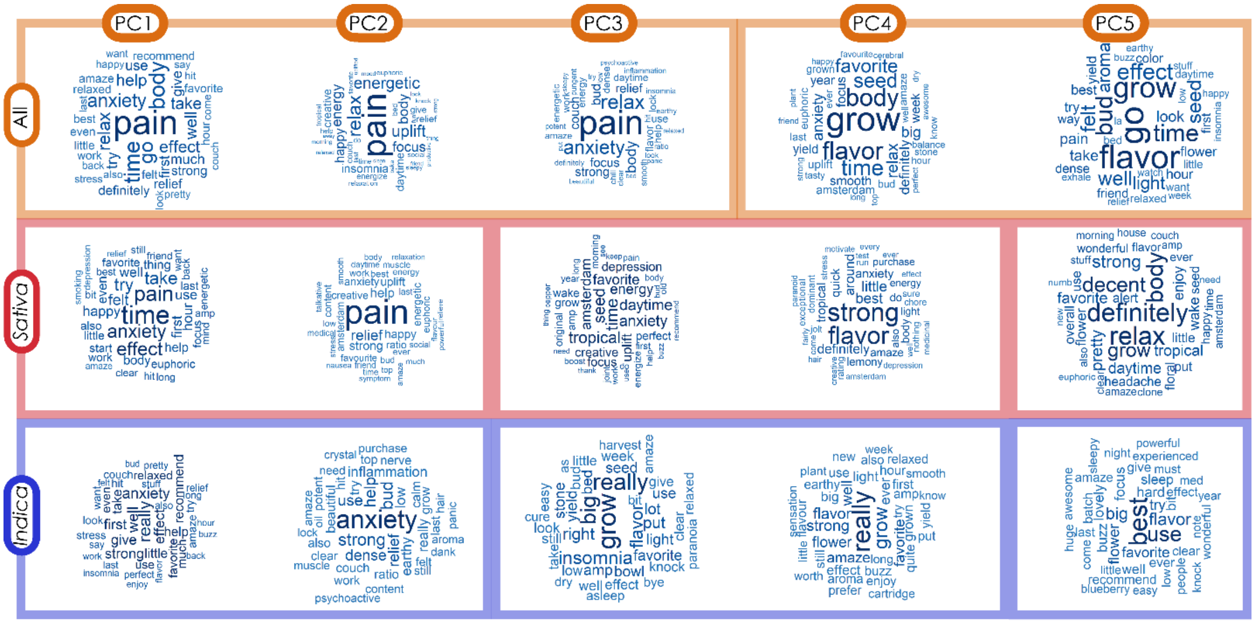

Supplementary Figure 7 Topics

Word clouds representing topics extracted with PCA from the term-document matrix. The first, second and third rows present topics for all cultivars combined, "sativas", and "indicas", respectively. Vertical lines are based on visual inspection and separate the content of the topics between subjective/therapeutic effects, and plant growth/acquisition.

5. Graphs community stability

Even though there is no clear threshold for Q values, Clauset et al. state that "a value above about 0.3 is a good indicator of significant community structure in a network" (Clauset et al., 2004). Most of our scores are below this threshold, which is why we refrain from drawing quantitative conclusions from this analysis. However, we find that in all cases the detected communities are stable, resulting in the same modules when repeating the process over multiple runs with random seeds for the Louvain algorithm. The lowest Q values we obtained correspond to LSA between all reports (Figure 4) which could result from the large number of shared words within the corpus (also evident in the word clouds). Supplementary table 5 shows the results obtained after 10 iterations using random seeds for the Louvain algorithm as implemented in Gephi. We would like to stress that we did not draw quantitative conclusions from the modular structure of these networks, using them only to aid the qualitative discussion, as well as for graphical support".

Supplementary Table 5 Modular stability

Supplementary Table 5 Modular stability

Q: Modularity values (Clauset et al., 2004); sd: standard deviation

Network	Q (mean/std)	N of modules (mean/sd)	Stability of presented modules
Strains by effects vectors (Figure 1A)	0.2647(0.0005)	17.5(0.7)	Yes
Strains by flavour vectors (Figure 2A)	0.220(0.002)	19.0(0.6)	Yes
LSA all reports (Figure 4)	0.0575(0.0007)	15.0(0.4)	Yes
Cannabinoids similarity (Figure 7A)	0.041(-)	8(-)	Yes
Terpenoids similarity (Figure 7B)	0.2461(0.0007)	2.8(0.4)	Yes
Flavours by terpenoids (Figure 7C)	0.3204(0.007)	3(-)	Yes

Effects by terpenoids (Figure 7C)	0.190(0.003)	3(-)	Yes

6. Bibliography

Bertoldo, A., Sparacino, G., Cobelli, C., 2004. “Population” approach improves parameter estimation of kinetic models from dynamic PET data, IEEE Transactions on Medical Imaging. https://doi.org/10.1109/TMI.2004.824243

Blondel, V.D., Guillaume, J.L., Lambiotte, R., Lefebvre, E., 2008. Fast unfolding of communities in large networks. J. Stat. Mech. Theory Exp. https://doi.org/10.1088/1742-5468/2008/10/P10008

Bonini, S.A., Premoli, M., Tambaro, S., Kumar, A., Maccarinelli, G., Memo, M., Mastinu, A., 2018. Cannabis sativa: A comprehensive ethnopharmacological review of a medicinal plant with a long history. J. Ethnopharmacol. 227, 300–315. https://doi.org/10.1016/j.jep.2018.09.004

Clauset, A., Newman, M., Moore, C., 2004. Finding community structure in very large networks. Phys. Rev. E 70, 1–6. https://doi.org/10.1103/PhysRevE.70.066111

De Lathauwer, L., De Moor, B., Vandewalle, J., 2000. An introduction to independent component analysis. J. Chemom. 14, 123–149. https://doi.org/10.1002/1099-128X(200005/06)14:3<123::AID-CEM589>3.0.CO;2-1

Dorogovtsev, S.N., Mendes, J.F.F., 2004. The shortest path to complex networks 25.

Friston, K.J., Poline, J.B., Holmes, a P., Frith, C.D., Frackowiak, R.S., 1996. A multivariate analysis of PET activation studies. Hum. Brain Mapp. 4, 140–51. https://doi.org/10.1002/(SICI)1097-0193(1996)4:2<140::AID-HBM5>3.0.CO;2-3

Gao, W., Zhu, H., Giovanello, K., 2009. Evidence on the emergence of the brain’s default network from 2-week-old to 2-year-old healthy pediatric subjects. PNAS.

Gilbert, A.N., DiVerdi, J.A., 2018. Consumer perceptions of strain differences in Cannabis aroma. PLoS One 13, 1–14. https://doi.org/10.1371/journal.pone.0192247

Hastie, T.T., 2009. The Elements of Statistical Learning. Math. Intell. 27, 83–85. https://doi.org/10.1007/b94608

Hillig, K.W., 2004. A chemotaxonomic analysis of terpenoid variation in Cannabis. Biochem. Syst. Ecol. 32, 875–891. https://doi.org/10.1016/j.bse.2004.04.004

Hillig, K.W., Mahlberg, P.G., 2004. A chemotaxonomic analysis of cannabinoid variation in Cannabis (Cannabaceae). Am. J. Bot. 91, 966–975. https://doi.org/10.3732/ajb.91.6.966

Huang, T.S., Narendra, P.M., 2008. Image restoration by singular value decomposition. Appl. Opt. https://doi.org/10.1364/ao.14.002213

James, G., Witten, D., Hastie, T., Tibshirani, R., 2013. An Introduction to Statistical Learning, Synthesis Lectures on Mathematics and Statistics, Springer Texts in Statistics. Springer New York, New York, NY. https://doi.org/10.1007/978-1-4614-7138-7

Jikomes, N., Zoorob, M., 2018. The Cannabinoid Content of Legal Cannabis in Washington State Varies Systematically Across Testing Facilities and Popular Consumer Products. Sci. Rep. 8, 1–15. https://doi.org/10.1038/s41598-018-22755-2

LAMARCK, J., 1785. Encyclopédique méthodique, Botanique I (part 2): 694–695. Panckoucke, Paris, Fr.

Lambiotte, R., Delvenne, J.-C., Barahona, M., 2008. Laplacian Dynamics and Multiscale Modular Structure in Networks 1–29. https://doi.org/10.1109/TNSE.2015.2391998

Landauer, T.K., Foltz, P.W., Laham, D., 1998. An introduction to latent semantic analysis. Discourse Process. 25, 259–284. https://doi.org/10.1080/01638539809545028

Lewis, M.A., Russo, E.B., Smith, K.M., 2018. Pharmacological Foundations of Cannabis Chemovars. Planta Med. 84, 225–233. https://doi.org/10.1055/s-0043-122240

Naganawa, M., Kimura, Y., Ishii, K., 2005. Extraction of a plasma time-activity curve from dynamic brain PET images based on independent component analysis. Biomed. … 52, 201–210.

Piomelli, D., Russo, E.B., 2016. The Cannabis sativa Versus Cannabis indica Debate: An Interview with Ethan Russo, MD. Cannabis Cannabinoid Res. 1, 44–46. https://doi.org/10.1089/can.2015.29003.ebr

Rong, X., 2014. word2vec Parameter Learning Explained 1–21.

Supekar, K., Menon, V., Rubin, D., Musen, M., Greicius, M.D., 2008. Network Analysis of Intrinsic Functional Brain Connectivity in Alzheimer's Disease. PLoS Comput. Biol. 4, 11.

Tipping, M., Bishop, C., 1999. Mixtures of probabilistic principal component analyzers. Neural Comput.
